# Localization and Phylogenetic Analysis of Enzymes Related to Organellar Genome Replication in the Unicellular Rhodophyte *Cyanidioschyzon merolae*

**DOI:** 10.1093/gbe/evu009

**Published:** 2014-01-09

**Authors:** Takashi Moriyama, Naoyuki Tajima, Kohsuke Sekine, Naoki Sato

**Affiliations:** ^1^Department of Life Sciences, Graduate School of Arts and Sciences, The University of Tokyo, Japan; ^2^JST, CREST, K's Gobancho, Tokyo, Japan

**Keywords:** evolution of replication apparatus, mitochondria and plastids, subcellular localization, GFP-fusion proteins, red algae

## Abstract

Plants and algae possess plastids and mitochondria harboring their own genomes, which are replicated by the apparatus consisting of DNA polymerase, DNA primase, DNA helicase, DNA topoisomerase, single-stranded DNA maintenance protein, DNA ligase, and primer removal enzyme. In the higher plant *Arabidopsis thaliana*, organellar replication-related enzymes (OREs) are similar in plastids and mitochondria because many of them are dually targeted to plastids and mitochondria. In the red algae, there is a report about a DNA replicase, plant/protist organellar DNA polymerase, which is localized to both plastids and mitochondria. However, other OREs remain unclear in algae. Here, we identified OREs possibly localized to organelles in the unicellular rhodophyte *Cyanidioschyzon merolae*. We then examined intracellular localization of green fluorescent protein-fusion proteins of these enzymes in *C. merolae*, whose cell has a single plastid and a single mitochondrion and is suitable for localization analysis, demonstrating that the plastid and the mitochondrion contain markedly different components of replication machinery. Phylogenetic analyses revealed that the organelle replication apparatus was composed of enzymes of various different origins, such as proteobacterial, cyanobacterial, and eukaryotic, in both red algae and green plants. Especially in the red alga, many enzymes of cyanobacterial origin remained. Finally, on the basis of the results of localization and phylogenetic analyses, we propose a model on the succession of OREs in eukaryotes.

## Introduction

Plants and algae possess plastids and mitochondria. Both organelles contain their own genome to perform their metabolic functions. The organellar genomes are thought to be replicated by the machinery including DNA polymerase, DNA primase, DNA helicase, DNA topoisomerase, single-stranded DNA binding protein (SSB), DNA ligase, and primer removal enzyme (Langston et al. 2009). As plant organellar genomes do not encode these enzymes, the organellar genomes must be replicated by the enzymes that are encoded by the nuclear genome and transported to the organelles after their synthesis (Wang et al. 1997).

In *Escherichia coli*, DNA polymerase III (Pol III) holoenzyme consisting of ten subunits functions in DNA elongation in replication. The catalytic α subunit (encoded by the *dnaE* gene) of Pol III belongs to Family-C DNA polymerase, and it is conserved in all bacteria (Langston et al. 2009; Sanyal and Doig 2012). DnaB helicase unwinds double-stranded DNA and then SSB maintains the DNA unwound. DNA gyrase, which consists of two subunits, gyrase A and B, is a type II DNA topoisomerase, and alleviates the strain resulting from DNA unwinding. DnaG synthesizes an RNA primer at the origin of replication in the leading strand and synthesizes primers in about every 1 kb in the lagging strand. Ribonucleotides within the RNA primer are removed by nick translation with 5′-3′ exonuclease and polymerase activity of DNA polymerase I (Pol I), and the nicked DNA is sealed by NAD^+^-dependent DNA ligase LigA.

In humans, the mitochondrial genome is replicated by a replisome consisting of DNA polymerase γ (Polγ), human mitochondrial RNA polymerase (POLRMT), TWINKLE helicase (T7 gp4-like protein with intramitochondrial nucleoid localization), topoisomerases 1 and 3a, SSB, ligase 3, and RNase H1 (Arnold et al. 2012; Kasiviswanathan et al. 2012). Polγ is responsible for the replication and repair in animal mitochondrial genomes, and belongs to Family-A DNA polymerase, which shares distant sequence similarity to bacterial Pol I. Animal Polγ comprises two subunits: a large subunit with DNA polymerase and 3′-5′ exonuclease activities, and a small subunit that enhances processivity and primer recognition (Kaguni 2004). POLRMT is a homolog of the RNA polymerase of T3/T7 phage (RPOT), which is a single polypeptide enzyme. POLRMT had been thought to function in transcription, but recently this RNA polymerase was demonstrated to function also as a primase (Wanrooij et al. 2008). In plants, the homolog of POLRMT is called RPOT type, and RPOT was localized to plastids and/or mitochondria for transcription (Kühn et al. 2007). TWINKLE is a homolog of the gp4 protein of T7 phage, which has helicase and primase activities. TWINKLE is widely conserved in eukaryotes including animals, bikonts (plants and protists), and amoebozoa (Shutt and Gray 2006). Animal TWINKLE shows only helicase activity and retains a nonfunctional domain of primase. Mitochondrial replisome was reconstituted with Polγ, TWINKLE, and SSB, and the resulting replisome showed rolling-circle replication with high processivity (Korhonen et al. 2004). Processivity is defined as the number of nucleotides added by a DNA polymerase per one binding to the template DNA, and in general, replicative DNA polymerase has a high processivity value, and repair polymerase has a low processivity value.

Recently, in higher plant *Arabidopsis thaliana*, enzymes related to organellar genome replication have been identified. The genome of *Arabidopsis* encodes two genes, each encoding a DNA polymerase belonging to Family-A DNA polymerase, which are called Pol I-like or Polγ (Christensen et al. 2005; Mori et al. 2005; Parent et al. 2011; Cupp and Nielsen 2013). These polymerases function in replication in both plastids and mitochondria. Subsequently, we reported that these polymerases are also conserved in many protists, and the enzymes are phylogenetically distinct from bacterial Pol I and Polγ. Because it was thought that a new name is needed, we proposed to call them POP (plant/protist organellar DNA polymerase, Moriyama et al. 2011; Moriyama and Sato 2013). *Arabidopsis thaliana* has a single gyrase A that is localized to both plastids and mitochondria, and has two gyrases B1 and B2 that are localized to chloroplasts and mitochondria, respectively. Inhibitor assay and complement test in *E. coli* revealed that *A. thaliana* gyrases are involved in organellar genome replication (Wall et al. 2004). *Arabidopsis thaliana* has an A-type topoisomerase I (AtTOP1), which is a homolog of bacterial topoisomerase I, TopA. Localization analysis using green fluorescent protein (GFP) fusion protein showed dual localization of AtTOP1 to chloroplasts and mitochondria (Carrie et al. 2009). In addition to this report, by proteome analysis, AtTOP1 was detected in the chloroplast nucleoids in *A. thaliana* (Olinares et al. 2010). *Arabidopsis thaliana* TWINKLE is localized to both chloroplasts and mitochondria (Carrie et al. 2009), and has both helicase and primase activities unlike animal TWINKLE retaining only helicase activity (Diray-Arce et al. 2013). Recombinant *Arabidopsis* SSB (AtSSB) binds to single-stranded DNA (ssDNA), but not to double-stranded DNA, and AtSSB-GFP protein is localized to mitochondria (Edmondson et al. 2005). Organellar SSB (OSB), which has a SSB-like domain, is a plant-specific SSB. *Arabidopsis thaliana* has four OSBs: AtOSB1 and 2 are localized to mitochondria, chloroplasts, respectively, and AtOSB3 is localized to both chloroplasts and mitochondria (Zaegel et al. 2006). Replication protein A (RPA), a nucleus-localized SSB in eukaryotes, comprises three subunits, RPA70, RPA32, and RPA14. Rice has three RPA70s, three RPA32s, and one RPA14. These subunits make three types of complexes, namely, type A, B, and C complexes with different combinations of the three types of subunits. Among them, type A RPA complex is localized to chloroplasts in rice (Ishibashi et al. 2006). DNA ligase 1 (LIG1) is localized to both mitochondria and nucleus, but not to plastids, and plastidial ligase remains unclear (Sunderland et al. 2006). In bacteria, Pol I acts in primer removal with its 5′-3′ exonuclease activity. The *Arabidopsis* genome encodes two 5′-3′ exonuclease genes (5′-3′EXO1 and 2) having sequence homology to 5′-3′ exonuclease domain of bacterial Pol I, and these enzymes were predicted to be localized to chloroplasts or mitochondria (Sato et al. 2003).

Previously, we demonstrated that a rhodophyte *Cyanidioschyzon merolae* has two organellar DNA polymerases, POP and Pol I (Moriyama et al. 2008). By immunoblot analysis using isolated organelles and observation of GFP-fusion proteins in onion epidermal cells, we showed that CmPOP was localized to both plastids and mitochondria, while CmPol I was localized to plastids. We also determined enzymatic activity of these polymerases. CmPOP showed the high processivity value (>1,300 nt) and had 3′-5′ exonuclease activity, while CmPol I showed middle level processivity (<70 nt), and had no 3′-5′ exonuclease activity (Moriyama et al. 2008). Other organellar replication-related enzymes (OREs) have not been identified in red and green algae.

The unicellular rhodophyte *C. merolae* has a remarkably simple cell structure consisting of a single mitochondrion and a single plastid per cell. Its normal habitat is hot springs, which is warm (up to 50 °C) and acidic (pH 1.5–2.5) with sulfuric acid. The size of its completely sequenced genome is 16,546,747 bp, with 4,775 predicted protein-coding genes (Matsuzaki et al. 2004; Nozaki et al. 2007). The method of transformation by polyethylene glycol (PEG) was recently established in *C. merolae* (Ohnuma et al. 2008). Subcellular localization of GFP-fusion protein in *C. merolae* was first demonstrated by Watanabe et al. (2011). They constructed the pCG1 vector, in which the target protein-*GFP* fusion gene is overexpressed by *C. merolae apcC* promoter.

In the present study, we identified the components of OREs, and analyzed their organellar localization by observation of GFP-fusion protein. We also estimated the origin of OREs by phylogenetic analysis. Finally, we discuss a model on the succession of organellar replisome in eukaryotes.

## Materials and Methods

### Culture Conditions

Cells of *C. merolae* strain 10D (Toda et al. 1998) were inoculated in the 2 × Allen’s medium (Minoda et al. 2004) at pH 2.5. Flasks were shaken under continuous light provided by two fluorescent tubes (30 µmol/m^2^/s) at 40 °C.

### Construction of GFP-Fusion Genes

Genes of putative OREs were amplified by PCR with specific primer sets (supplementary table S1, Supplementary Material online). Using In-Fusion HD cloning kit (Clontech Laboratories, Mountain View, CA), the amplified DNA fragments were inserted into *Xba*I-cut pCG1 vector (Watanabe et al. 2011) that contains the *apcC* gene promoter of *C. merolae*, sGFP, and *NOS* terminator.

### Transformation in *C. merolae* Cells

Cells of *C. merolae* were transformed by the PEG-method according to Ohnuma et al. (2008) except for the preparation of recipient cells. Subcultured cells were grown to OD_750_ = 3–5 with aeration by 1% CO_2_, and collected by centrifugation at 4,000 × g for 5 min at 40 °C. The precipitated cells were resuspended in 2 × Allen's medium to OD_750_ = 11–12. The concentrated culture was subjected to darkness for 12 h with aeration by 1% CO_2_ at 40 °C in a special culture apparatus that was assembled from materials for gel electrophoresis. Namely, two 0.8-mm silicone tubes (0.8-mm wall, Bio-Rad Laboratories, Hercules, CA) were inserted between the two glass plates with a silicone spacer (clearance of 2 mm) for aeration (supplementary fig. S1, Supplementary Material online). The cells were grown in this apparatus under high light provided by two 20-W krypton bulbs (250 µmol/m^2^/s) for 12–16 h. To 100 μl of the culture taken from the apparatus was added to 150 μl of a DNA mixture (15 μl of 10 × MA-I [Ohnuma et al. 2008], 100 μg of salmon sperm DNA, and 50 μg of plasmid DNA), and then 250 μl of 60% PEG solution in MA-I was added to the cell–DNA mixture. Three minutes later, the mixture was diluted to 40 ml with 2 × Allen's medium, and cultured overnight at 40 °C with aeration by 1% CO_2_.

### Microscopic Examination

For observation of fluorescence of GFP, transformed cells were collected by centrifugation at 200×g for 10 min, and examined by a fluorescence microscope BX-60 (Olympus, Tokyo, Japan), using the cube U-MNIBA (Olympus). When GFP fluorescence was faint, we performed immunofluorescence detection using anti-GFP antibody, essentially according to Nishida et al. (2004). In the reaction with antibody, an anti-GFP monoclonal antibody was diluted to 1/200 with immunoreaction enhancer solution (Can Get Signal immunostain solution B, TOYOBO, Osaka, Japan) and reacted for 1 h. As the secondary antibody, anti-mouse monoclonal antibody labelled with Alexa fluor 488 (Invitrogen, Carlsbad, CA) was diluted to 1/200 with immunoreaction enhancer solution, and reacted for 30 min. The immunostained cells were observed by the fluorescence microscope with the cube U-MNIBA (Olympus) for Alexa fluor 488, the cube U-MWIG (Olympus) for observation of autofluorescence of chlorophyll, and the cube U-MWU (Olympus) for observation of DAPI-stained DNA. Nomarski differential interference image was also recorded. All microscopic images were captured by a digital camera (model DP-70, Olympus).

### Phylogenetic Analysis

Sequence data of homologous proteins of *C. merolae* OREs were obtained from the Gclust database (Sato 2009, version 2010-05, data set Gclust2010e29b). The sequence of T7 gp4 was obtained from Genbank. Amino acid alignments were prepared by the MUSCLE software version 3.6 (Edgar 2004). Manipulation of sequence data were performed by the SISEQ software version 1.59 (Sato 2000). The alignments were used to infer phylogeny by the Maximum Likelihood method using the TreeFinder software version March 2011 (Jobb et al. 2004) with the WAG model with “Empirical” option.

## Results

### Replication-Related Genes Whose Products Are Possibly Localized to Organelles in *C. merolae* Genome

Table 1 is a list of putative components of OREs in *C. merolae*, selected by Gclust database search (Sato 2009) using replication enzymes in bacteria and OREs in higher plants and animals as references (supplementary table S2, Supplementary Material online). *Cyanidioschyzon merolae* genome encoded homologs of most OREs of *A. thaliana*, except OSB, which is conserved only in higher plants (Zaegel et al. 2006). *Cyanidioschyzon merolae* has a putative DnaG primase (encoded by the nuclear genome) and DnaB helicase (encoded by the plastid genome), which are bacterial enzymes that are not encoded by the genomes of higher plants and animals. *Cyanidioschyzon merolae* also has a homolog of bacterial Pol I harboring two domains, 5′-3′ exonuclease and DNA polymerase, while green plants have an enzyme containing a single 5′-3′ exonuclease domain. *Cyanidioschyzon merolae* has two eukaryotic topoisomerase II, CMB013C and CML330C, which we named TOP2a and TOP2b.

**Table 1:** List of Replication-Related Enzymes Possibly Localized to Plastid or Mitochondrion in *C. merolae* and Summary of Localization Analysis

Annotation	Gene Identifier	Phylogenetic Origin	Prediction Results			GFP
			TargetP	PSORT	Predotar	
DNA polymerase						
POP	CMO270C	Unknown	mt	pt	ER	pt, mt
Priming						
DnaG	CMQ286C	Cyanobacterial	mt	mt	None	pt
DNA helicase						
DnaB (pt-encoded)	CMV098C	Cyanobacterial				(pt^a^)
Primase/helicase						
TWINKLE	CMT452C	Eukaryotic	***mt***	pt	***mt***	mt
Topoisomerases						
Gyrase A	CMS243C	Cyanobacterial	None	***pt***	ER	pt
Gyrase B	CMH166C	Cyanobacterial	***pt***	***pt***	None	pt
Topoisomerase I (type IA)	CMI252C	Cyanobacterial	***pt***	***pt***	None	pt
Topoisomerase I (type IB)	CMM263C	Eukaryotic	None	nuc	None	nuc
Topoisomerase II a	CMB013C	Eukaryotic	pt	pt	None	cyto^b^
Topoisomerase Il b	CML330C	Eukaryotic	***mt***	cyto	None	mt
Topoisomerase III alpha	CMG056C	—	mt	pt	mt	—
Topoisomerase VI A-1	CME071C	—	None	pt	None	—
Topoisomerase VI A-2	CML010C	—	mt	pt	None	—
Topoisomerase VI A-3	CMQ111C	—	mt	pt	None	cyto^b^
Topoisomerase VI A-4	CMR274C	—	mt	pt	None	—
Topoisomerase VI B	CMT273C	—	None	nuc	None	—
ssDNA maintenance						
SSB	CMI135C	α-Proteobacterial	***mt***	***mt***	***mt***	mt
RPA 70 kDa	CMC123C	—	mt	pt	mt	nuc
RPA 30 kDa	CMI291C	—	None	nuc	None	nuc
Ligation						
DNA ligase	CMK235C	Eukaryotic	mt	pt	None	pt, mt
Primer removal						
DNA Pol I	CMT462C	α-Proteobacterial	mt	***pt***	mt	pt
RNase HI large subunit	CMK297C	—	None	nuc	None	—
RNase HII	CMT626C	Bacterial (unknown origin)	***mt***	pt	***mt***	mt
DNA2	CMK133C	—	mt	pt (nuc)	None	nuc
FEN1	CMG106C	—	None	pt	mt	nuc

Note.—Predicted results of localization consistent with the GFP results are marked by bold italic. Abbreviations: pt, plastid; mt, mitochondrion; nuc, nucleus; cyto, cytosol; ER, endoplasmic reticulum.

^a^No GFP analysis was done because *dnaB* gene is encoded by the plastid genome.

^b^In this study, N-terminal peptides were fused with GFP for targeting analysis, and cytosol localization was observed in these enzymes. However, these enzymes have a nuclear localization signal within the protein sequence after the N-terminus.

Three computer programs, TargetP (http://www.cbs.dtu.dk/services/TargetP/, last accessed January 22, 2014), WolfPSORT (http://www.genscript.com/psort/wolf_psort.html, last accessed January 22, 2014), and Predotar (http://urgi.versailles.inra.fr/predotar/predotar.html, last accessed January 22, 2014) were used for the prediction of subcellular localization of putative OREs. The results were considerably different among the three programs (table 1). We also made alignments of putative OREs and determined whether putative OREs have an N-terminal extension for organellar targeting (supplementary fig. S2-1 to S2-9, Supplementary Material online). On the basis of these results, we selected proteins for analysis of subcellular targeting.

### Intracellular Localization of Putative OREs in *C. merolae*

**Fig. 1.—:**
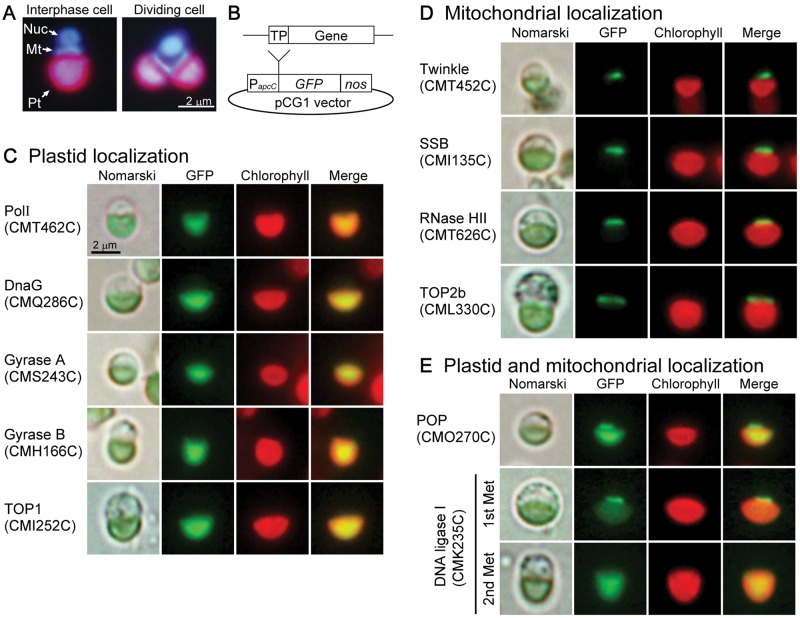
Localization of ORE-GFP fusion proteins in *C. merolae* cell. (*A*) Fluorescence images with DAPI staining of an interphase cell and a dividing cell. Nuc; Nucleus, Mt; Mitochondrion, Pt; Plastid. (*B*) Schematic representation of the constructs expressing GFP fusion proteins under the control of the *apcC* promoter of *C. merolae* (P*apcC*) and the *NOS* terminator (*nos*). Inserts corresponding to transit peptide regions (TP) were cloned into the pCG1 vector. (*C–E*) Fluorescence microscopic images of the transiently transformed *C. merolae* cells. Images of OREs localized to plastid (*C*), mitochondrion (*D*), or both plastid and mitochondrion (*E*) are shown.

The *C. merolae* cell has a simple cell structure with a single plastid and a single mitochondrion, and reproduces itself by binary fission (fig. 1*A*). The N-terminal extension sequence of each putative ORE was cloned into pCG1 vector for overexpression of its GFP-fusion protein driven by the *apcC* promoter (fig. 1*B*). The amino acid sequences of putative OREs fused with GFP are given in supplementary figure S2, Supplementary Material online. The plasmids were introduced into *C. merolae* cells by the PEG method, and GFP fluorescence was observed (fig. 1*C*–*E* and supplementary fig. S3, Supplementary Material online). When GFP fluorescence in the nucleus-like structure was faint, the protein targeting was confirmed by immunostaining using anti-GFP antibody with 4′,6-diamidino-2-phenylindole (DAPI) staining (supplementary fig. S3, Supplementary Material online).

The DNA replicase, POP, was dually targeted to plastid and mitochondrion (fig. 1*E*), which is consistent with our previous results by immunoblotting (Moriyama et al. 2008). There are two methionine residues in the N-terminus region of LIG1 (supplementary fig. S2-1, Supplementary Material online). A construct starting from the first methionine residue in LIG1 showed strong GFP-fluorescence in the mitochondrion and weak fluorescence in the plastid, whereas another construct starting from the second methionine residue showed GFP-fluorescence in the plastid (fig. 1*E*). LIG1 might be translated from the two different methionine residues.

TWINKLE, which had helicase/primase activity and was localized to both plastids and mitochondria in *A. thaliana*, was observed only in mitochondrion in *C. merolae* (fig. 1*D*). DnaG primase, which was conserved in red algae and not in green plants, was localized to plastid (fig. 1*C*). In *C. merolae*, DnaB helicase is encoded by the plastid genome, and the enzyme is supposed to be localized to plastid. Among DNA topoisomerases, two subunits (A and B) of gyrase and type-IA topoisomerase I (TOP1) were localized to the plastid, while topoisomerase II-b (TOP2b) was localized to the mitochondrion (fig. 1*C* and *D*). Other tested topoisomerases, namely, TOP1 (type IB), TOP2A, and TOP6, were not observed in either plastid or mitochondrion (supplementary fig. S3, Supplementary Material online). The *C. merolae* genome encodes five enzymes related to primer removal, RNase HI and HII, DNA2, FEN1, and Pol I that has 5′-3′ exonuclease and polymerase domains. The GFP-fusion results suggested that Pol I and RNase HII are localized to plastid and mitochondrion, respectively (fig. 1*C* and *D*). DNA2 and FEN1 were observed in the nucleus in *C. merolae* (supplementary fig. S3, Supplementary Material online).

The fluorescence of SSB-GFP was observed only in the mitochondrion (fig. 1*D*). We also tested a construct starting from the second methionine residue in SSB, and the construct showed nonorganellar localization (data not shown). In rice, an RPA was detected in isolated plastids, by immunoblotting, as well as in nuclei (Ishibashi et al. 2006). However, *C. merolae* RPAs have no extension sequence at its N-terminus, and these were localized to the nucleus (Supplementary fig. S3, Supplementary Material online). No other gene for ssDNA binding protein is found in the *C. merolae* genome. RNA binding protein may function also as a ssDNA binding protein. It is known that some RNA binding proteins, such as TIA-1 and TIAR in humans, bind to ssDNA (Suswam et al. 2005).

To examine localization of enzymes translated from native start codon(s) including non-AUG start codon, we first observed using constructs, authentic promoter:full-length protein:GFP. However, no fluorescent signal of these constructs was observed even if immunofluorescence using anti-GFP antibody was performed. Next, we prepared constructs overexpressed by *apcC* promoter, namely, *apcC* promoter:full-length protein:GFP. Also in these constructs except SSB, GFP-fluorescence could not be observed. Overexpressed SSB-GFP was observed as a single granule in mitochondrion, not in nucleus in *C. merolae* cell (data not shown). This granular structure might occur by artifact with overexpression of full-length SSB. Subcellular localization of other full-length OREs could not be determined yet, and we do not discard the possibility that some of the proteins we found in this study that went to only mitochondria, plastids, or the nucleus could still be dual-localized.

### Phylogenetic Analysis of OREs

Excepting some clearly eukaryotic enzymes, such as POP, type IB and IIB topoisomerases, and DNA ligase I, we analyzed the phylogenetic origin of OREs. With respect to the POP, we previously reported that the origin of POP was unclear because POP did not originate from Pol I of cyanobacteria nor α-proteobacteria (Moriyama et al. 2008).

**Fig. 2.—:**
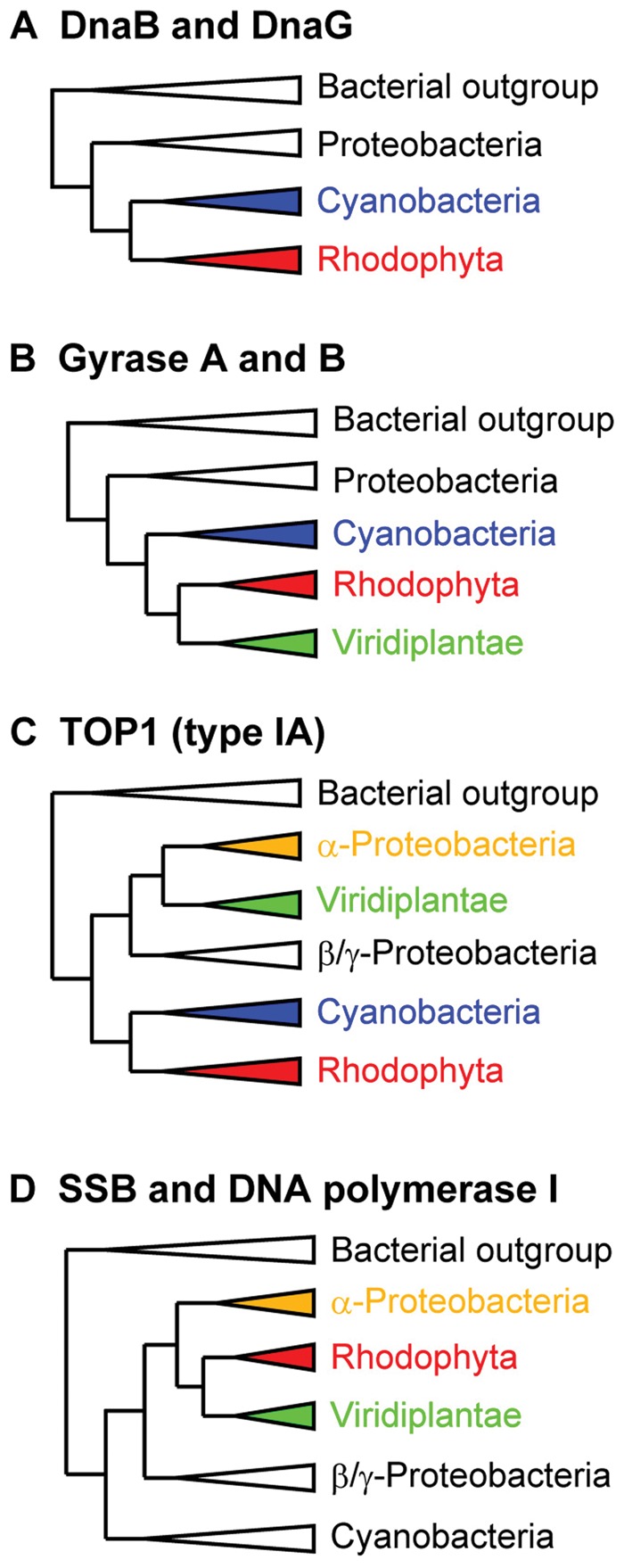
Simplified phylogenetic tree of OREs originating from bacteria. In rhodophytes, enzymes of cyanobacterial origin are DnaB, DnaG (*A*), gyrases (*B*), and TOP1 (*C*). Enzymes of α-proteobacterial origin are SSB and Pol I (*D*). Detailed phylogenetic trees are shown in supplementary figure S4, Supplementary Material online.

Four types of simplified trees of OREs are shown in figure 2, and original trees are also shown in supplementary figure S4-1 to S4-6, Supplementary Material online. DnaG and DnaB, which are not conserved in green plants, originated from cyanobacteria (fig. 2*A* and supplementary fig. S4-1, Supplementary Material online). Gyrases A and B originated from cyanobacteria in red algae and green plants (fig. 2*B* and supplementary fig. S4-2, Supplementary Material online). Type IA topoisomerase originated from cyanobacteria in red algae, but the enzyme was obviously related to α-proteobacterial homologs in green plants (fig. 2*C* and supplementary fig. S4-3, Supplementary Material online). Red algal and green plant SSB originated from the SSB in α-proteobacteria (fig. 2*D*). Animal SSB is also α-proteobacterial origin (supplementary fig. S4-4, Supplementary Material online). Because green plants have 5′-3′ exonuclease but not a full-domain of Pol I consisting of 5′-3′ exonuclease, 3′-5′ exonuclease, and DNA polymerase domain, we examined phylogenetic relationship of the 5′-3′ exonuclease domain in bacteria and photosynthetic eukaryotes. The results showed that exonucleases in the red and green lineages are monophyletic, and their origin is α-proteobacterial exonuclease domain (fig. 2*D* and supplementary fig. S4-4, Supplementary Material online). Red algae and green plants have bacterial RNase HII, but their RNase HII was not related to RNase HII in α-proteobacteria nor cyanobacteria. The enzyme might have been acquired by lateral gene transfer from bacteria such as Chlamydiae, Firmicutes, or Chlorobi (supplementary fig. S4-5, Supplementary Material online). We also performed phylogenetic analysis of TWINKLE. The TWINKLE of *C. merolae* and diatoms are closely related to that of animals and ciliates rather than to that of plants. It was reported that animal TWINKLE does not have primase activity because some key amino acid residues for primase activity are not conserved in animal TWINKLE. In *C. merolae*, the key residues are conserved, and *C. merolae* TWINKLE is assumed to possess primase activity (Shutt and Gray 2006).

## Discussion

### Components of Replication Enzymes in Organelles

In the present study, we identified the intracellular localization of OREs in *C. merolae*, namely, six plastid-localized OREs, four mitochondrion-localized OREs, and two dually localized OREs. In *C.merolae*, ORE components of plastid differed significantly with those of mitochondrion except dually localized ones, POP and LIG1. This is in contrast with the situation in *A. thaliana*, in which components of OREs in plastids and mitochondria are nearly identical.

Dual localization of POP was found in both the red alga *C. merolae* (Moriyama et al. 2008) and the green plants, *A. thaliana* (Christensen et al. 2005) and tobacco (Ono et al. 2007). In contrast, localization of other OREs is variable in various species of red algae and green plants. POP consists of a single polypeptide and showed high processivity, although POP belongs to Family A-type DNA polymerase that has sequence similarity to *E. coli* Pol I having a middle-level processivity (Moriyama et al. 2008). POP also showed a high fidelity with its 3′-5′ exonuclease activity (Takeuchi et al. 2007). These properties of POP suggest that POPs are widely conserved organellar replicase in eukaryotes including plants, amoebozoa, alveolates, heterokonts, and discristates (Moriyama and Sato 2013).

TWINKLE is identified as a DNA helicase in human mitochondria (Spelbrink et al. 2001). In *A. thaliana*, AtTWINKLE showed dual activity, primase and helicase, and this TWINKLE was thought to be involved in the replication of genomes in plastids and mitochondria in green plants (Diray-Arce et al. 2013). In *C. merolae*, however, TWINKLE is targeted to only mitochondria. Instead, single domain enzymes originating from cyanobacteria, DnaB helicase and DnaG primase, are localized to the plastid. A rhodophyte *Porphyridium purpureum*, and diatoms *Thalassiosira pseudonana* and *Phaeodactylum tricornutum* also have a plastid-encoded DnaB and a nucleus-encoded DnaG (supplementary fig. S4-1, Supplementary Material online). We confirmed that *Porphyridium* DnaG was localized to plastid in transformed *C. merolae* cells (data not shown). These results suggest that rhodophytes and diatoms (NB: the latter is thought to originate from the secondary endosymbiosis involving a rhodophyte) retain DnaB and DnaG of cyanobacterial origin, and these enzymes function in plastids, whereas the TWINKLE consisting of helicase and primase domains functions in mitochondria.

*Cyanidioschyzon merolae* has 12 genes encoding various subunits of topoisomerases, among which gyrases and bacterial TOP1 are localized to plastids, while eukaryotic TOP2b is localized to mitochondria. Organellar localization of eukaryotic TOP2 has not been reported in plants. In *C. merolae*, the effects of a gyrase-specific inhibitor, nalidixic acid, were reported by two groups (Itoh et al. 1997; Kobayashi et al. 2009). In these reports, nalidixic acid arrests not only the replication of plastid genome but also that of mitochondrial and nuclear genomes. Considering the plastid localization of gyrases in *C. merolae*, these results may suggest that the arrest of plastid replication influences mitochondrial and nuclear replication by an unknown mechanism. In another possible explanation, nalidixic acid might inhibit mitochondrial and/or nuclear topoisomerases in *C. merolae*.

### Evolution of Enzymes Involved in Organellar Genome Replication in Photosynthetic Organisms

**Fig. 3.—:**
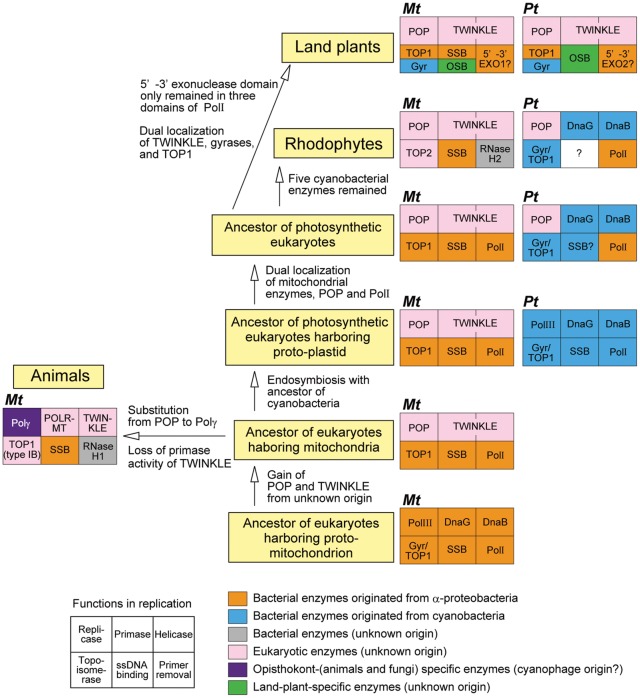
Schematic illustration of the processes leading to succession of OREs in eukaryotes. Boxes consisting of six small boxes indicate component enzymes of organellar replication, and explanatory notes for each location of boxes are shown at the lower left corner of this figure. Organelle names, mitochondria (Mt), and plastid (Pt), are indicated in the upper left corner of each box. The enzymes that originated from α-proteobacteria and cyanobacteria are shown in orange and blue boxes, respectively. Eukaryotic enzymes, whose origin is unclear, are shown in pink boxes. Polγ is conserved only in opisthokonts including animals and fungi, and the enzyme is shown in a purple box. Bacterial enzymes derived from unknown origin are shown in gray boxes. A land-plant-specific enzyme, OSB, is shown in green boxes. ssDNA binding protein in plastids of rhodophytes was not identified in the present study, and the box is indicated by a question mark.

Previously, we proposed a model for the succession of organellar DNA polymerase in eukaryotes (Moriyama et al. 2011; Moriyama and Sato 2013). In the present report, addition of other OREs completes the entire view of the model (fig. 3). First, when the ancestor of eukaryotes acquired mitochondria, the genome of the protomitochondrion had been replicated with the α-proteobacterial replication machinery, but then the host cell started to use a distinct set of replicase, helicase, and primase. Namely, Pol III was replaced by POP, and DnaB and DnaG were replaced by TWINKLE. The origins of POP and TWINKLE are still unknown. In animals, POP was replaced by Polγ whose origin is estimated as a T-odd phage DNA polymerase (Filée et al. 2002). In addition, a recent finding that some cyanophages have a homolog of Polγ suggests that Polγ might originate from a cyanophage enzyme (Chan et al. 2011). Moreover, in animals, TWINKLE lost primase activity and animal mitochondria started to use POLRMT (T3/T7 phage type RNA polymerase) for priming on replication in addition to transcription. SSB is a sole ORE of the α-proteobacterial origin in animals. After the second endosymbiosis that engendered the plastids in the common ancestor of Viridiplantae and Rhodophyta, cyanobacterial Pol III was replaced by POP, originally functioning in mitochondria. Plastids of rhodophytes continued to use DnaB and DnaG of cyanobacterial origin, while plastids of green plants started to use TWINKLE in place of DnaB and DnaG. Pol I of the α-proteobacterial origin was retained in photosynthetic eukaryotes; however, its 3′-5′ exonuclease and DNA polymerase domains were lost in land plants, and in rhodophytes, 3′-5′ exonuclease activity was lost.

Accordingly, in land plants, components of replication enzymes had been exchanged between plastids and mitochondria, and are now homogeneous in these organelles. In red algae, however, components of OREs are essentially different between plastid and mitochondrion. Plastid in red algae retains many OREs originating from cyanobacteria. Among these cyanobacteria-derived OREs, DnaB is encoded by the plastid genome, while others are encoded by the nuclear genome in red algae. The same is true in diatoms. The mechanism of replication in the plastid genome in the red lineages might need to retain plastid-encoded *dnaB* gene for helicase activity or its nucleotide sequence. Cooperative function of DnaB and DnaG in replication and protein–protein interaction of these enzymes have been reported in *E. coli* (Tougu and Marians 1996), and this relationship may be conserved in red lineages. In the next step of research, functional analysis of replication apparatus in each organelle will be important. Reconstitution experiments in vitro of the organellar DNA replication by a combination of various ORE proteins will provide clues for the problem.

## Supplementary Material

Supplementary tables S1, S2, and figures S1–S4 are available at *Genome Biology and Evolution* online (http://www.gbe.oxfordjournals.org/).

Supplementary Data
