# Supplementary material for: Localization and Phylogenetic Analysis of Enzymes Related to Organellar Genome Replication in the Unicellular Rhodophyte Cyanidioschyzon merolae
Source: Genome Biol Evol. 2014 Jan 9;6(1):228–37. doi: 10.1093/gbe/evu009 (PMC3914683; doi:10.1093/gbe/evu009)
Supplement: Supplementary Data [file supp_6_1_228__index.html]

Localization and phylogenetic analysis of enzymes related to organellar genome replication in the unicellular rhodophyte Cyanidioschyzon merolae — Localization and Phylogenetic Analysis of Enzymes Related to Organellar Genome Replication in the Unicellular Rhodophyte Cyanidioschyzon merolae — Supplementary Data 

# Localization and Phylogenetic Analysis of Enzymes Related to Organellar Genome Replication in the Unicellular Rhodophyte *Cyanidioschyzon merolae*

## Supplementary Data

files

**Files in this Data Supplement:**

- Supplementary Data - pdf file
