# Supplementary material for: Localization and Phylogenetic Analysis of Enzymes Related to Organellar Genome Replication in the Unicellular Rhodophyte Cyanidioschyzon merolae
Source: Genome Biol Evol. 2014 Jan 9;6(1):228–37. doi: 10.1093/gbe/evu009 (PMC3914683; doi:10.1093/gbe/evu009)
Supplement: Supplementary Data [file supp_evu009_Supplement_all.pdf]

**Supplementary material**

**Tables**

Table S1. List of primers used in the present study. The underlined sequences, which are required for cloning using In-Fusion reaction (Clontech), are the sequence of the ends of pCG1 vector linearized by *Xba*I. The number preceding F (forward primer) or R (reverse primer) in each primer name indicates the position of nucleotide starting from the initiation codon of each gene.

| Primer | Sequence (5' to 3') |
|---|---|
| CMB013C-1F | TTCGTTGACCTCTAGAATGAACACAAGCTCGGCGAG |
| CMB013C-171R | CCATGGATCCTCTAGAGGTATCGTCGAGCACCCACA |
| CMC123C-1F | TTCGTTGACCTCTAGAATGGCGAGCACAGCACTCAGTCGTG |
| CMC123C-1989R | CCATGGATCCTCTAGAGTGCAGCGCAGAAATGGTTTCTTGG |
| CMG106C-1F | TTCGTTGACCTCTAGAATGGGTATTCGACAGCTGGC |
| CMG106C-1140R | CCATGGATCCTCTAGAGGTTTTGCGTGCTCGTTTCG |
| CMH166C-1F | TTCGTTGACCTCTAGAATGAACGCCACTTATGCATTCGTGA |
| CMH166C-249R | CCATGGATCCTCTAGATGCGGCAGTGTACTCAGACCCAGAT |
| CMI135C-1F | TTCGTTGACCTCTAGAATGCAGGCCTCGATTCGTGCGCTAT |
| CMI135C-234R | CCATGGATCCTCTAGAAACGAGCAAAACACGATTAACGCTC |
| CMI252C-1F | TTCGTTGACCTCTAGAATGTGGGTAGGAGGTGCGTG |
| CMI252C-423R | CCATGGATCCTCTAGACGCCTTGCGGTTTCCAACTG |
| CMI291C-1F | TTCGTTGACCTCTAGAATGGACTATTCAAACACGTACGGCG |
| CMI291C-891R | CCATGGATCCTCTAGATGAGCTTGACGCTCGAAACGTATCA |
| CMK133C-1F | TTCGTTGACCTCTAGAATGGAGCGCTTTATTCAGCG |
| CMK133C-420R | CCATGGATCCTCTAGAAAACAATTTGCCTGGATACT |
| CMK235C-1F | TTCGTTGACCTCTAGAATGACCTGCAATTGGCAGCT |
| CMK235C-58F | TTCGTTGACCTCTAGAATGAAGGTTATCTGCACTGT |
| CMK235C-300R | CCATGGATCCTCTAGATCCTGTTCGGGCGTTCCAGG |
| CML330C-1F | TTCGTTGACCTCTAGAATGCGCTCACTAGGCGATAT |

| | |
|---|---|
| CML330C-171R | CCATGGATCCTCTAGAGCGGCGCTCGATTCTCTCGA |
| CMM263C-1F | TTCGTTGACCTCTAGAATGCATCAGCAGGACCCTGA |
| CMM263C-342R | CCATGGATCCTCTAGACGATTCATCTTCCGCTCTAT |
| CMO270C-130F | TTCGTTGACCTCTAGAATGCGGTTAGGCGTAGGGTCTTGCC |
| CMO270C-300R | CCATGGATCCTCTAGAAGCCGAACGGCGGGCGTTCGCACAC |
| CMQ111C-1F | TTCGTTGACCTCTAGAATGGTTGCGAAGAGAAAGAA |
| CMQ111C-300R | CCATGGATCCTCTAGACTTTGCTGCGGAGAGAATGA |
| CMQ286C-1F | TTCGTTGACCTCTAGAATGTCAGACGGAGCGACTTTGGGGA |
| CMQ286C-192R | CCATGGATCCTCTAGAGGCTACCAGTGCACGCCGAAAGCCC |
| CMS243C-1F | TTCGTTGACCTCTAGAATGCAGGAACTTGATTCTTACAAAC |
| CMS243C-393R | CCATGGATCCTCTAGAAATAATGCTTTCATGCGGGGAGGCT |
| CMT452C-1F | TTCGTTGACCTCTAGAATGACATACTGGAAAGATGGAGCGC |
| CMT452C-501R | CCATGGATCCTCTAGATACCATATGACCCGGGGGCAGTTCG |
| CMT462C-1F | TTCGTTGACCTCTAGAATGTTTGCAGTTTGCTGGTACTCAC |
| CMT462C-180R | CCATGGATCCTCTAGACCATTCGCACAGGTTCGTGTAACGA |
| CMT626C-1F | TTCGTTGACCTCTAGAATGGCGTATTTGGTCTCTCT |
| CMT626C-282R | CCATGGATCCTCTAGAACCGGCCAAGCGGAAGGCAT |

Table S2. Replication-related enzymes in *Escherichia coli*, *Arabidopsis thaliana* and *Homo sapiens*.

| | *E. coli*[a] | *A. thaliana* | | *H. sapiens*[k] |
|---|---|---|---|---|
| Organelles<br>Function | | Cp | Mt | Mt |
| DNA replicase | Pol III | POP[b] | POP[b] | Polγ |
| Priming | DnaG | TWINKLE[c,d] | TWINKLE[c,d] | POLRMT |
| DNA helicase | DnaB | TWINKLE[c,d] | TWINKLE[c,d] | TWINKLE |
| DNA topoisomerase | Gyr | Gyr[e], TOP1 (type IA)[c,f] | Gyr[e], TOP1 (type IA)[c] | TOP1 (type IB), TOP3a |
| ssDNA maintenance | SSB | OSB2[g] | SSB[h], OSB1,3[g] | SSB |
| Ligation | LigA | ? | LIG1[i] | LIG3 |
| RNA primer removal | Pol I | 5'-3' EXO1? (AT1G34380)[j] | 5'-3' EXO2? (AT3G52050)[j] | RNase H1 |

[a](Langston et al. 2009; Sanyal and Doig 2012), [b](Christensen et al. 2005; Parent et al. 2011), [c](Carrie et al. 2009), [d](Diray-Arce et al. 2013), [e](Wall et al. 2004), [f](Olinares et al. 2010), [g](Zaegel et al. 2006), [h](Edmondson et al. 2005), [i](Sunderland et al. 2006), [j](Sato et al. 2003), [k](Arnold et al. 2012; Kasiviswanathan et al. 2012).

**Figure legends**

Fig. S1. Picture of a culture vessel used in the present study for the preparation of *C. merolae* culture suitable for transformation.

Fig. S2. Alignments of OREs. The N-terminal sequence enclosed by a blue or orange box indicates the sequence fused with GFP in pCG1 vector. In GFP analysis, RPAs (30 and 70 kDa) and FEN1 were cloned into pCG1 vector with their full-length, because these enzymes have no N-terminal extension.

Fig. S3. Localization analysis of proteins that are not localized to organelles (plastid or mitochondrion). The localization was examined by observation of GFP-fluorescence or immunostaining (asterisked) using anti-GFP antibody with DAPI staining.

Fig. S4. Phylogenetic trees of OREs of bacterial origin and TWINKLE. Orange, blue, red and green characters show α-proteobacteria, cyanobacteria, red algae and plants, respectively. In the lower part of Figure S4-4, schematic comparison of the domain structure of Pol I and 5′-3′ exonuclease is shown. The colored boxes indicate domains estimated from the Pfam database: pink, 5′-3′ exonuclease domain; blue, 3′-5′ exonuclease domain; orange, DNA polymerase domain; gray, transit peptide sequence.

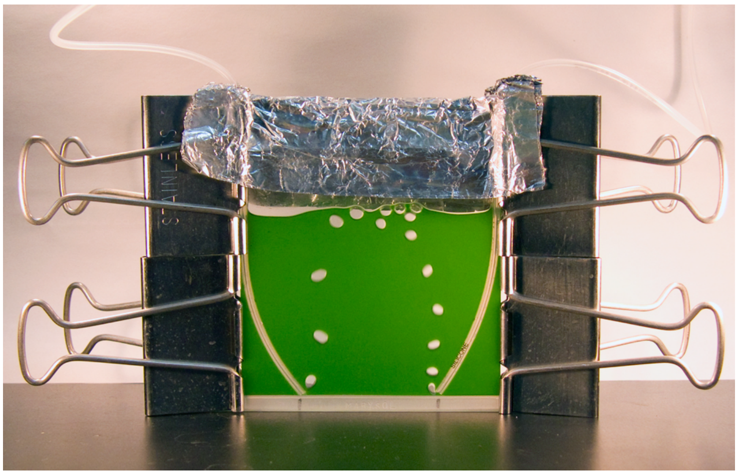

Supplementary Figure S1

## POP (plant/protist organellar DNA polymerase)

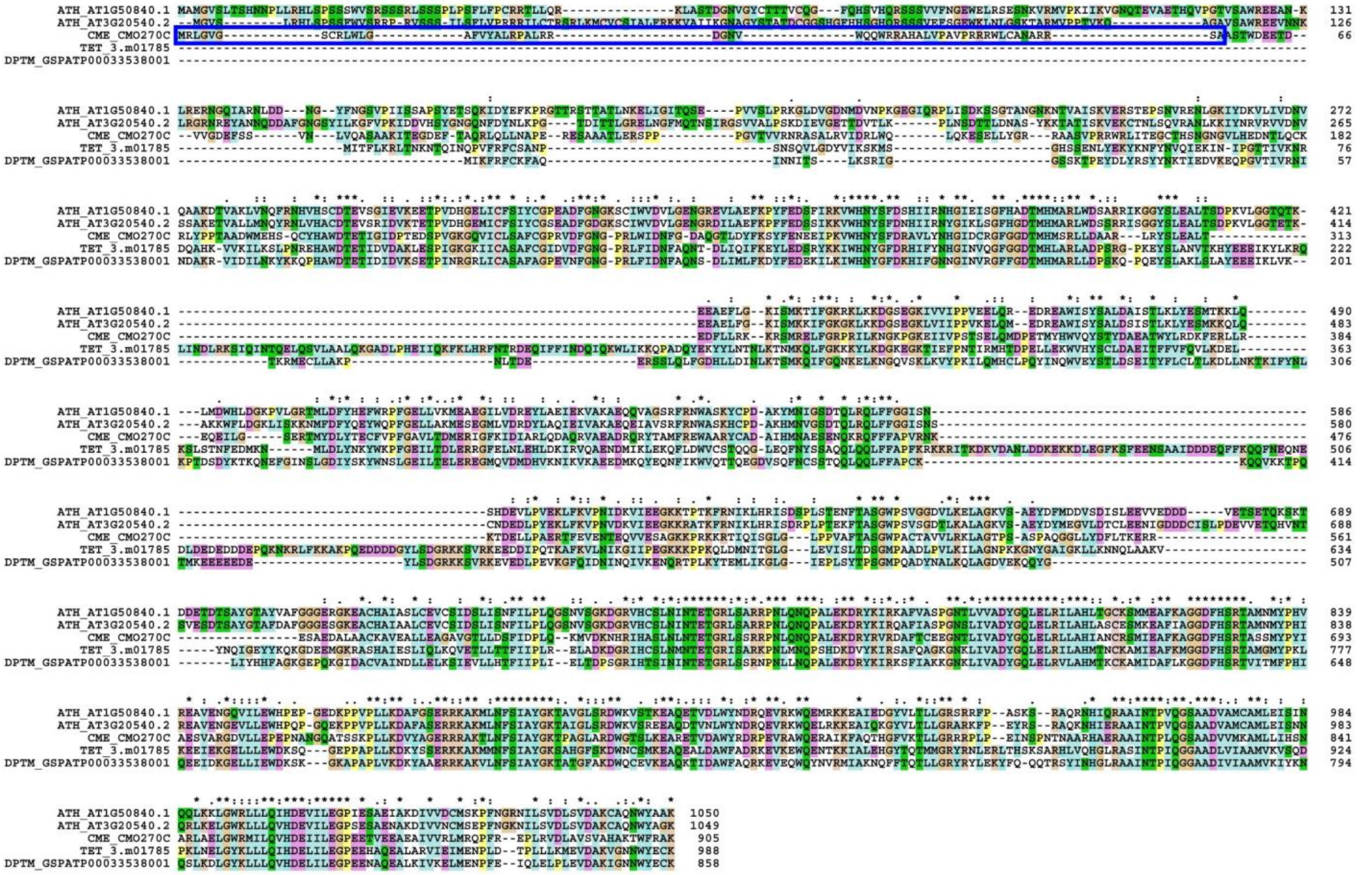

## DNA ligase I

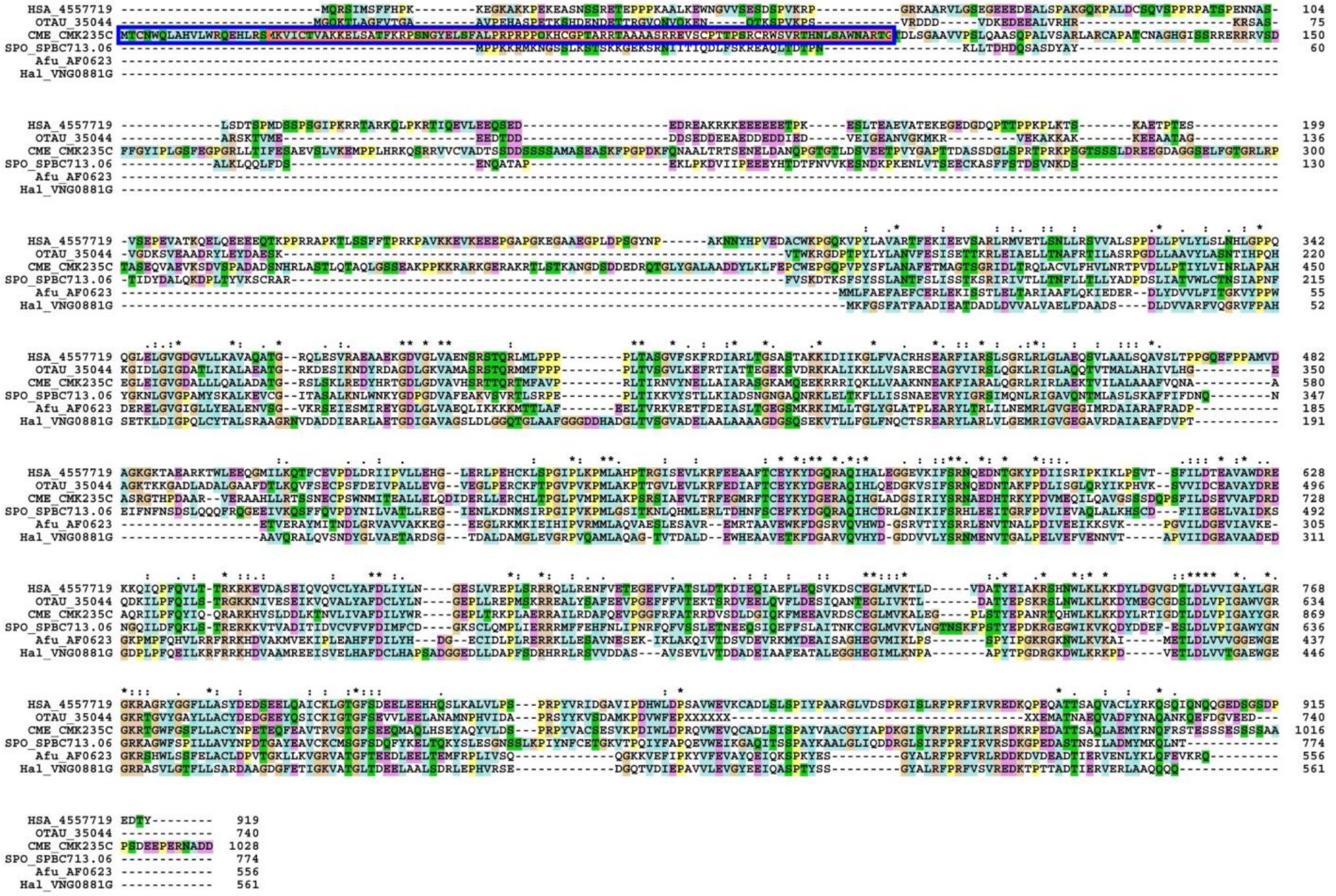

Supplementary figure S2-1

## DnaG primase

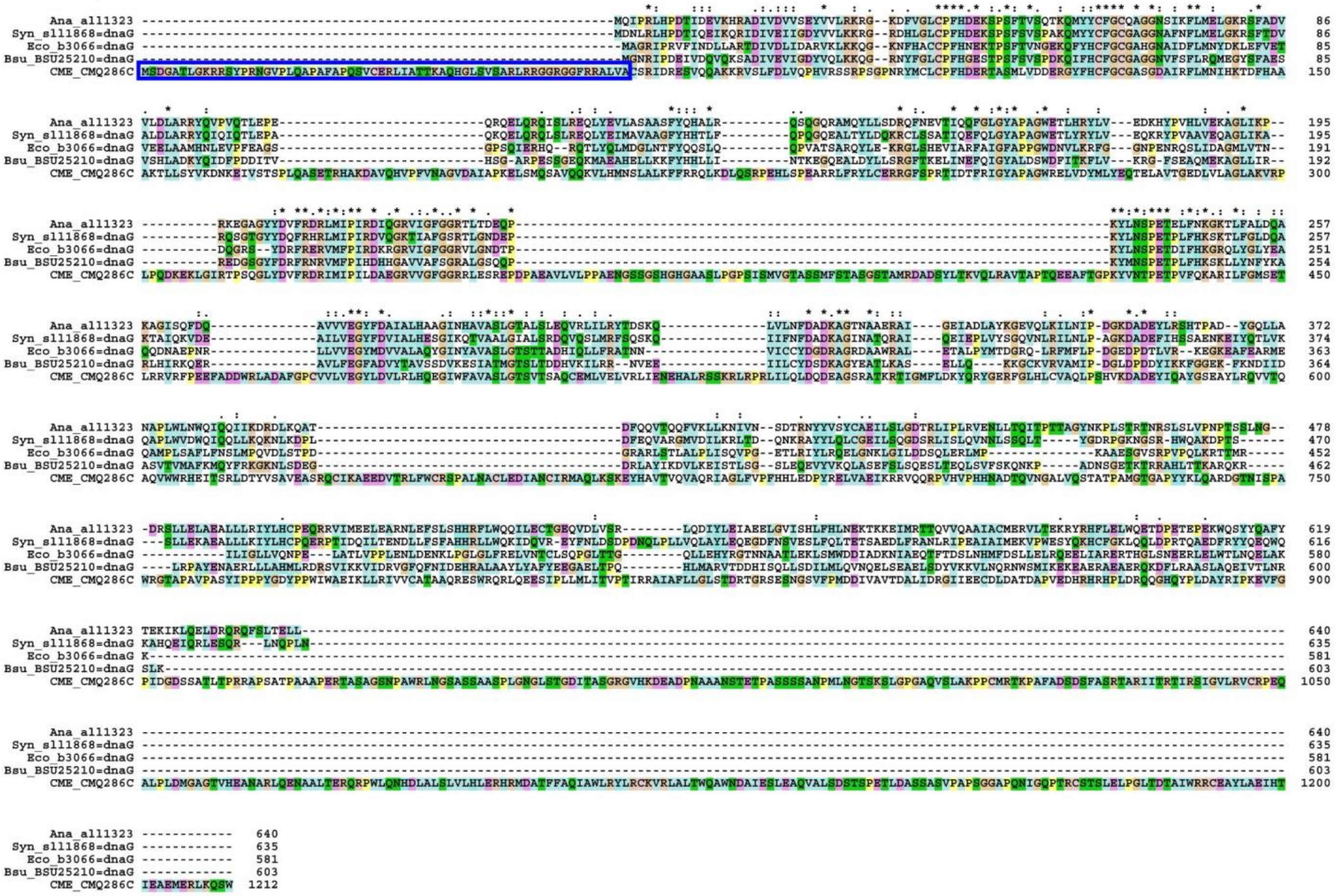

## Gyrase A

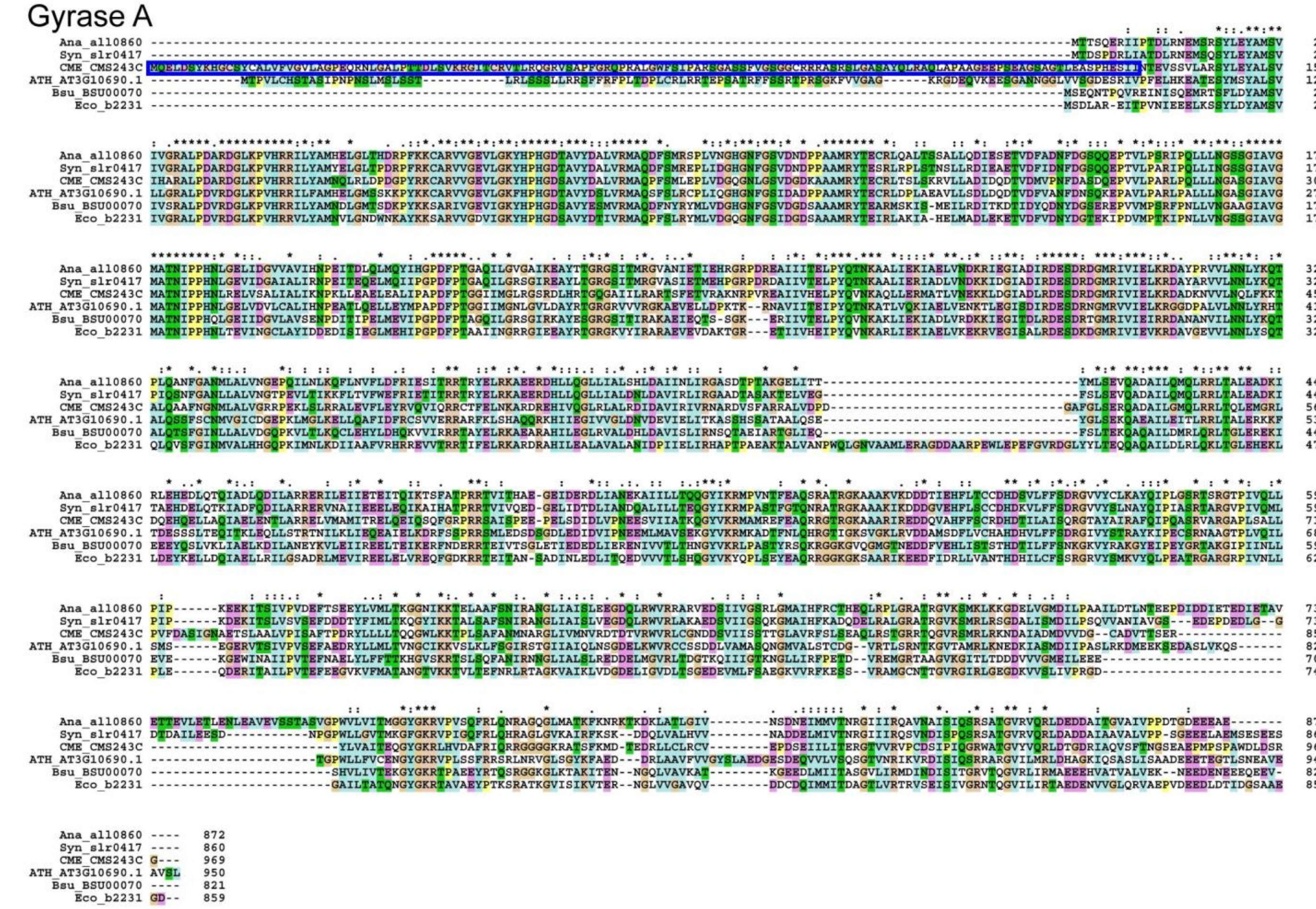

Supplementary figure S2-2

## Gyrase B

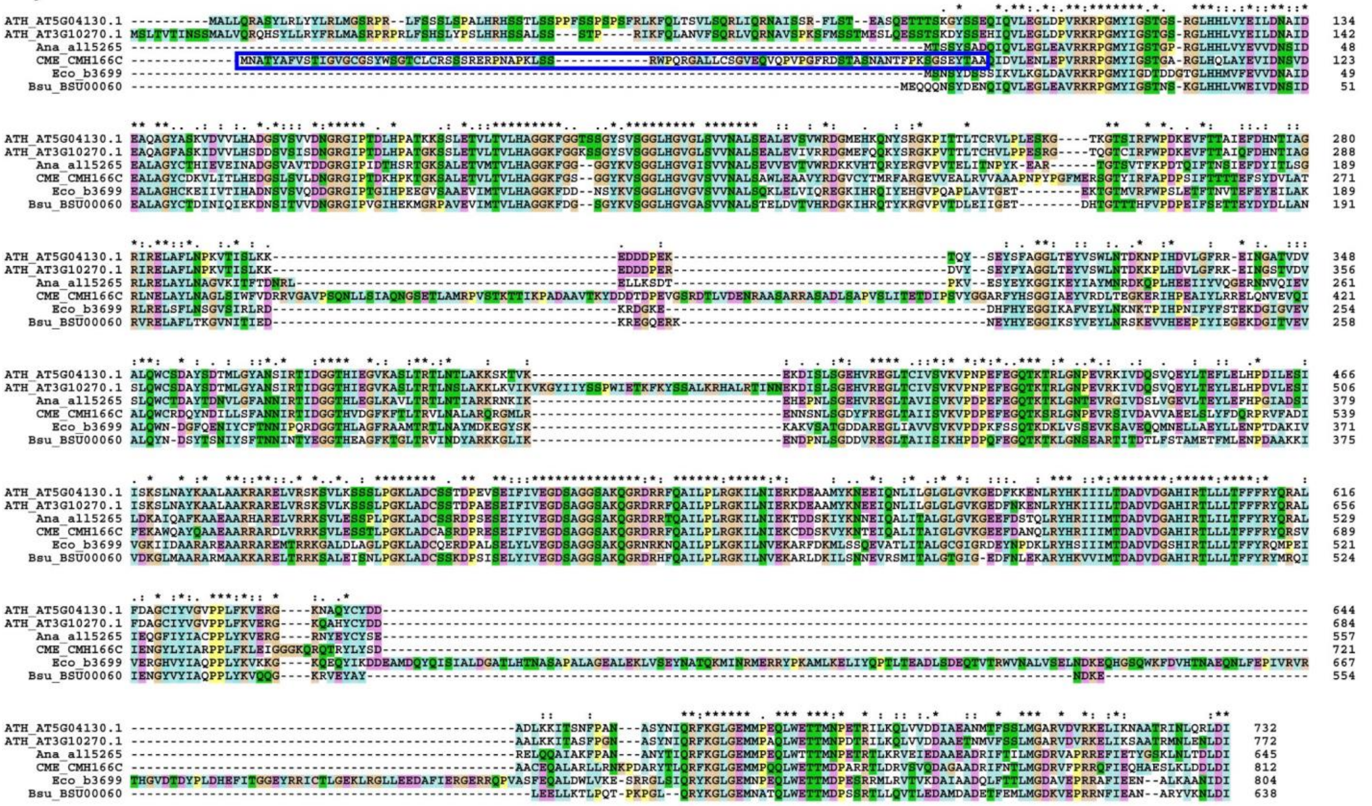

## DNA polymerase I

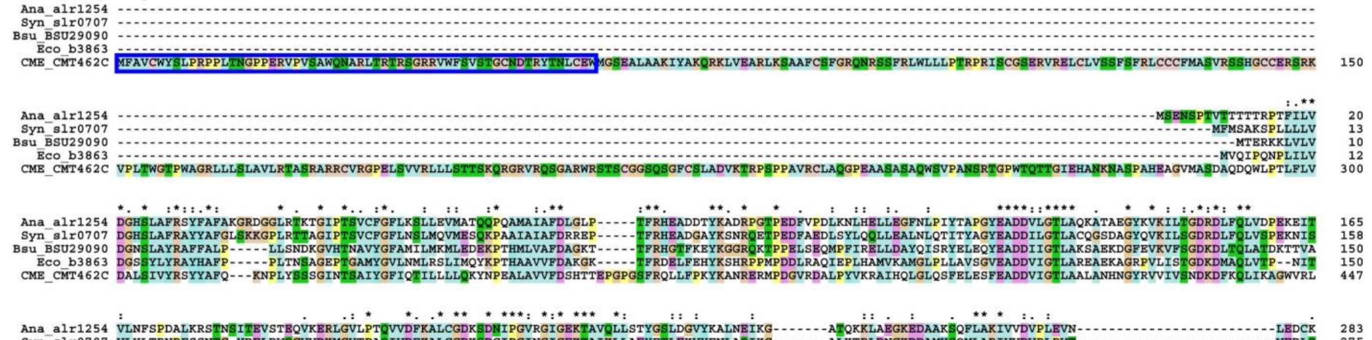

Supplementary figure S2-3

Topoisomerase I and III (type IA)

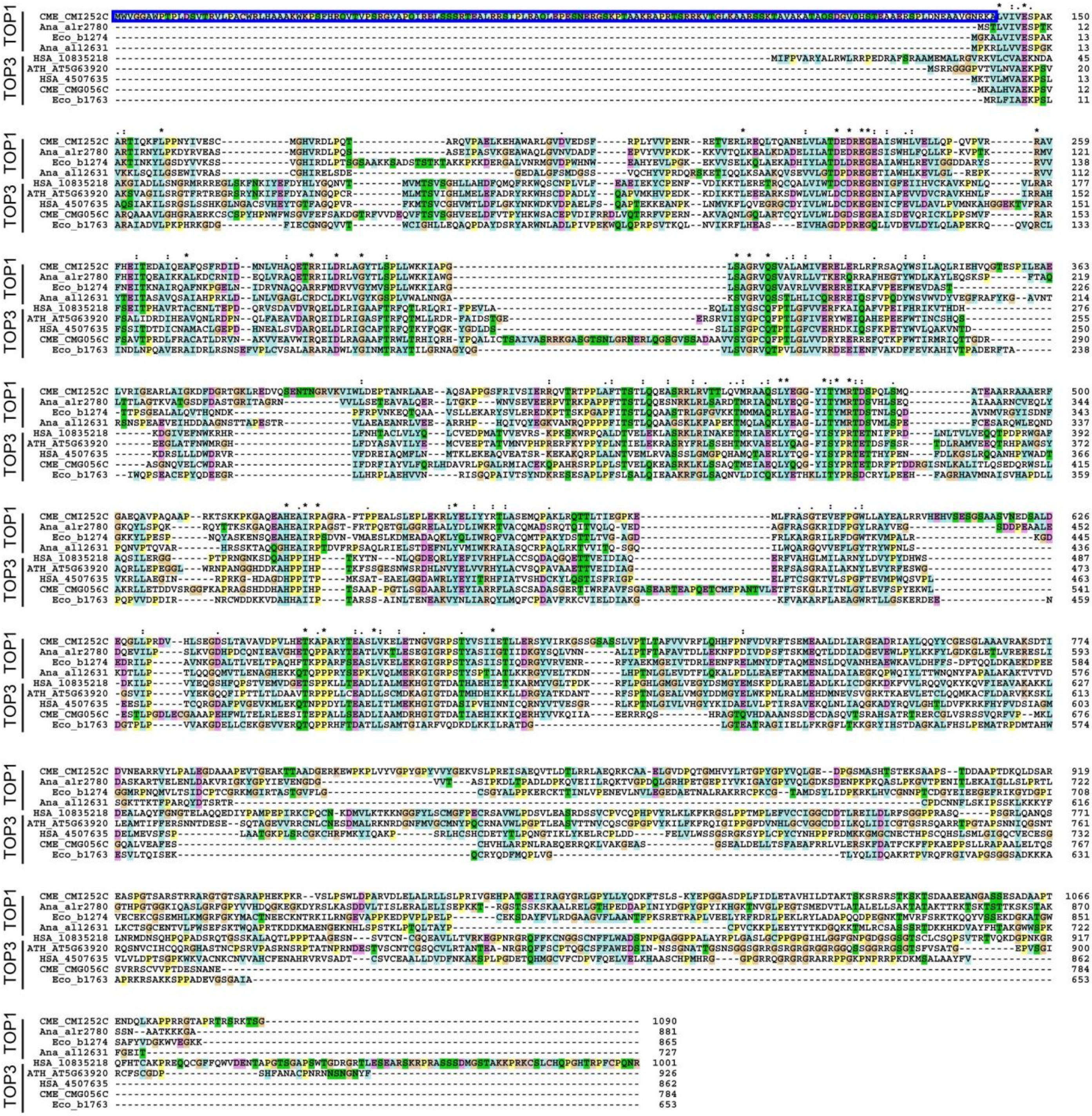


Supplementary figure S2-4

# TWINKLE

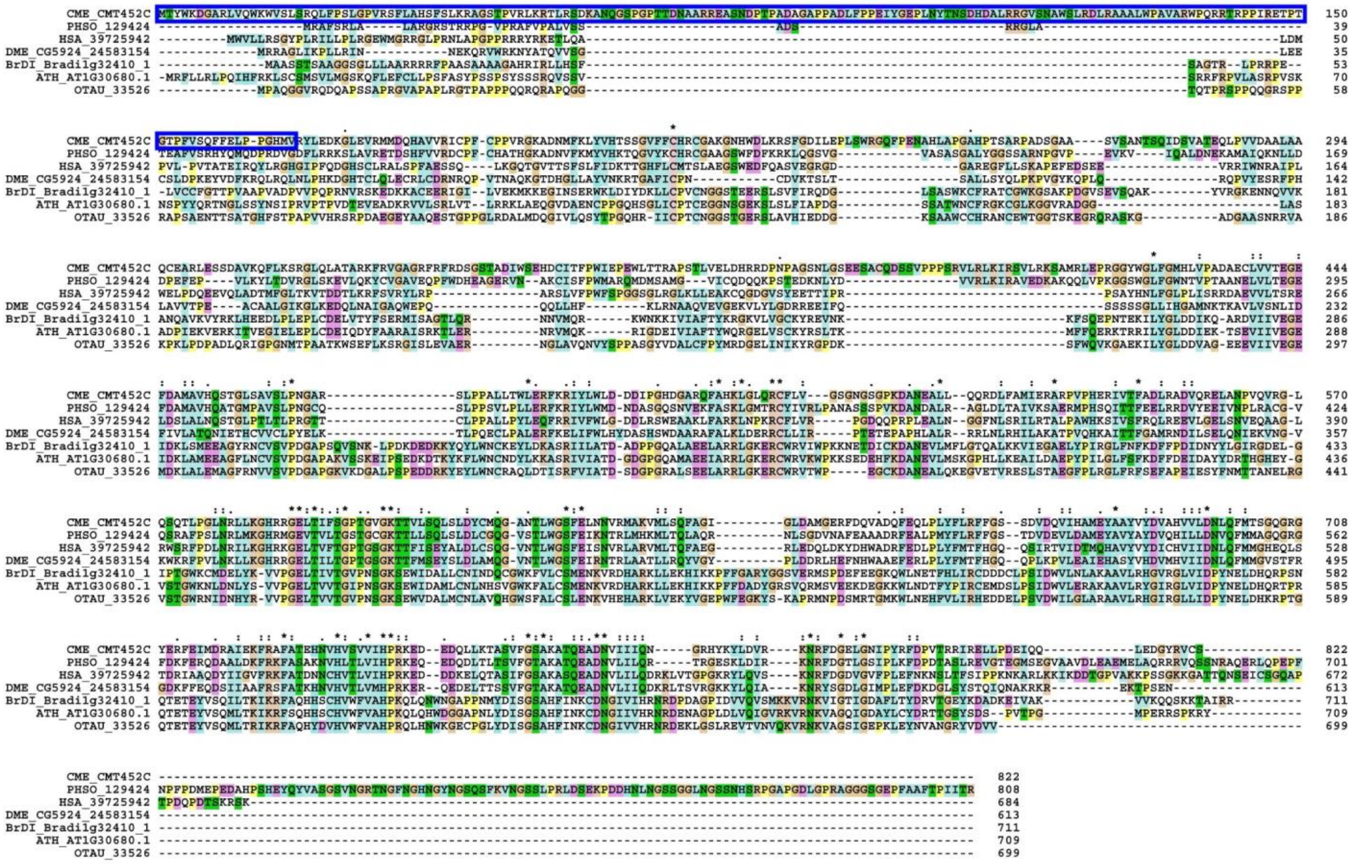


# SSB

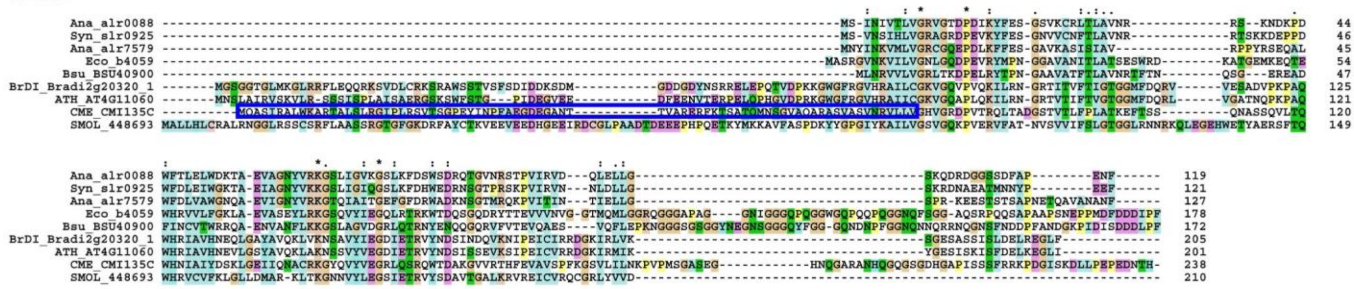


# RNase HII

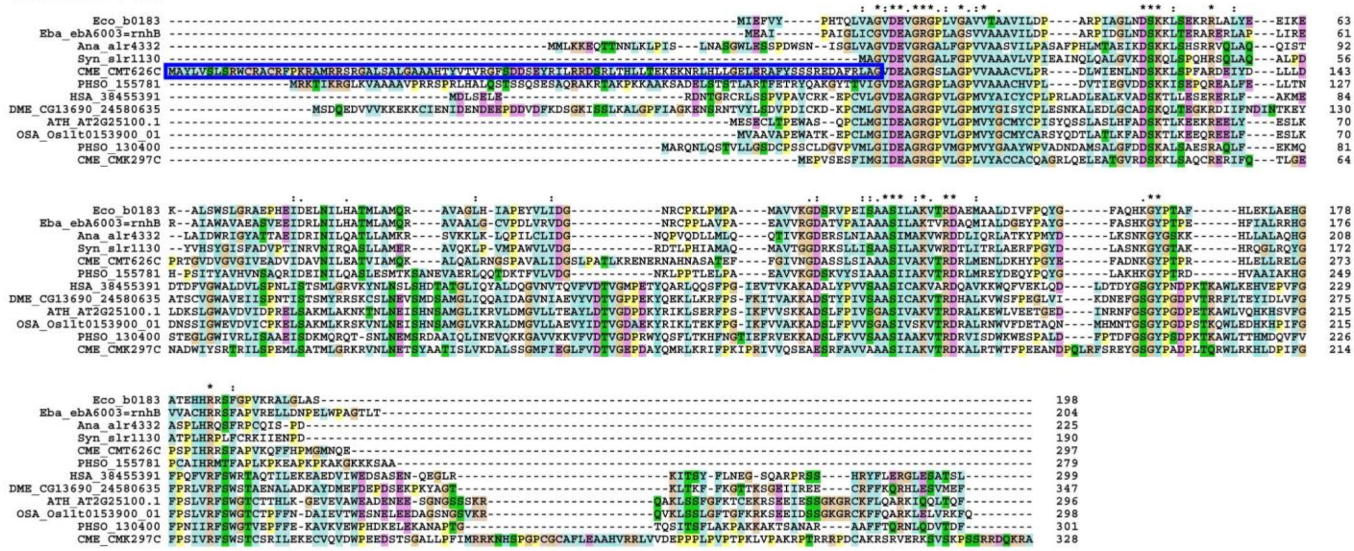


Supplementary figure S2-5

# Topoisomerase II

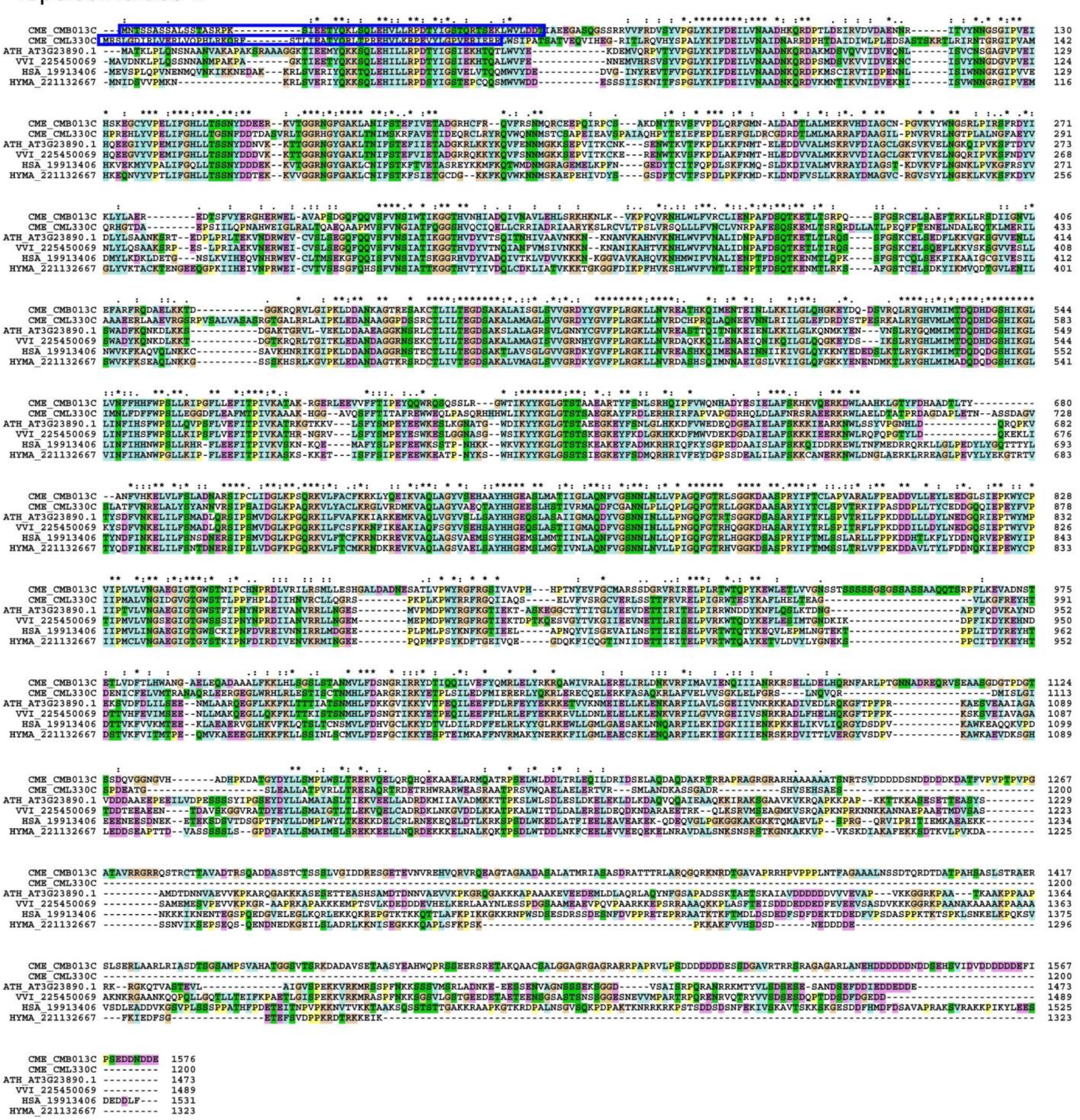

# Replication protein A 30 kDa

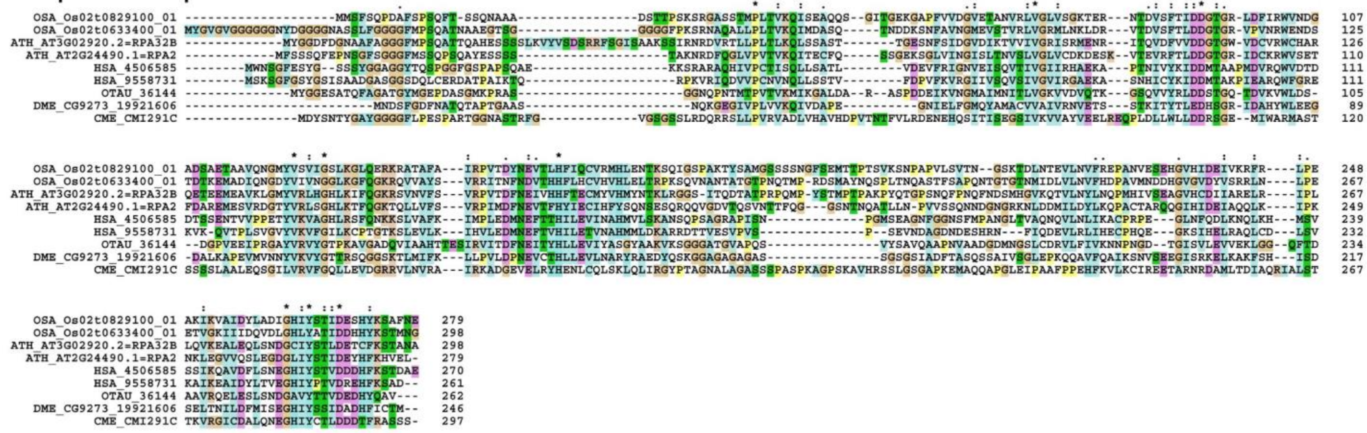

## Topoisomerase I (type IB)

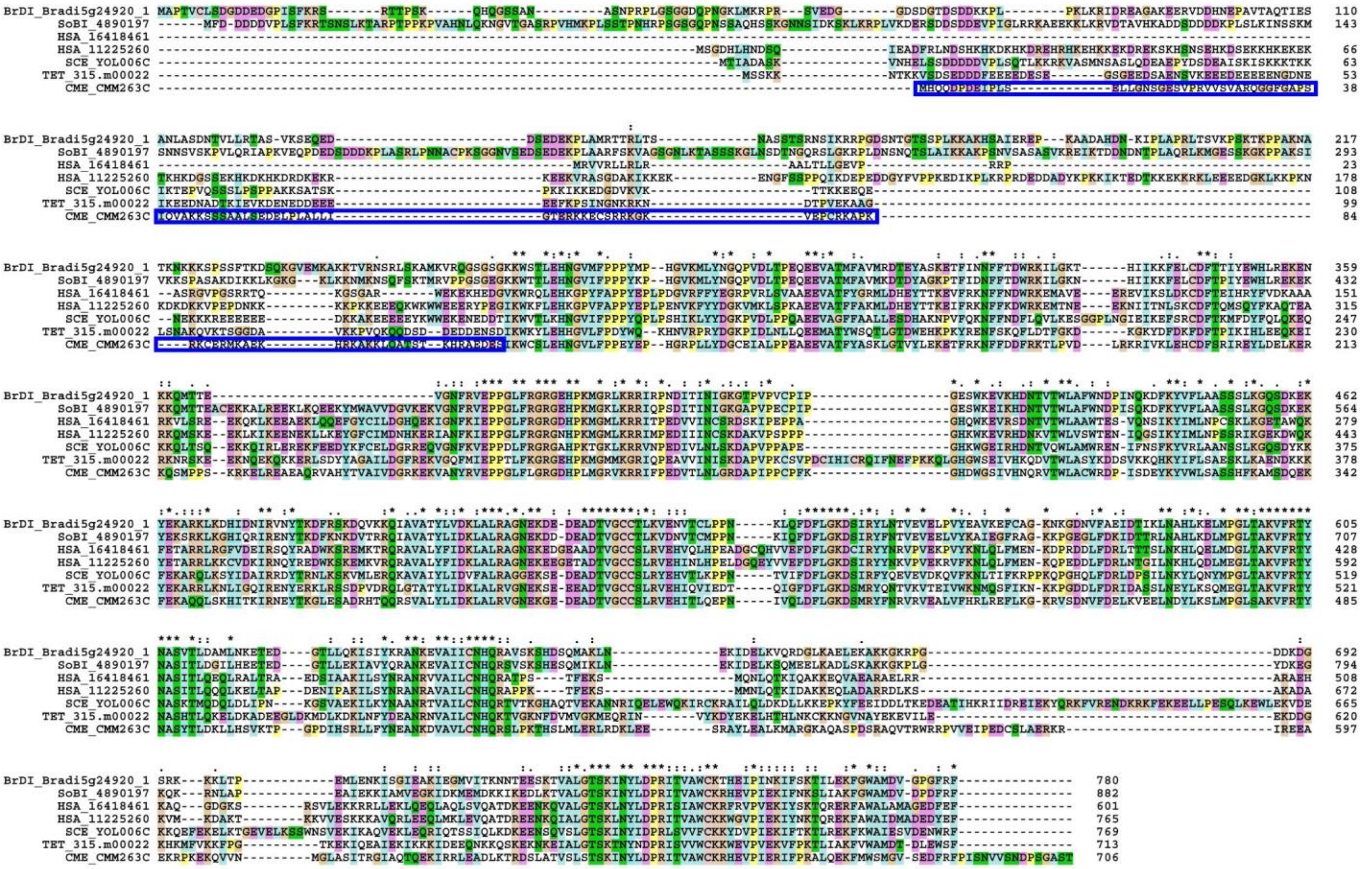

## Replication protein A 70 kDa

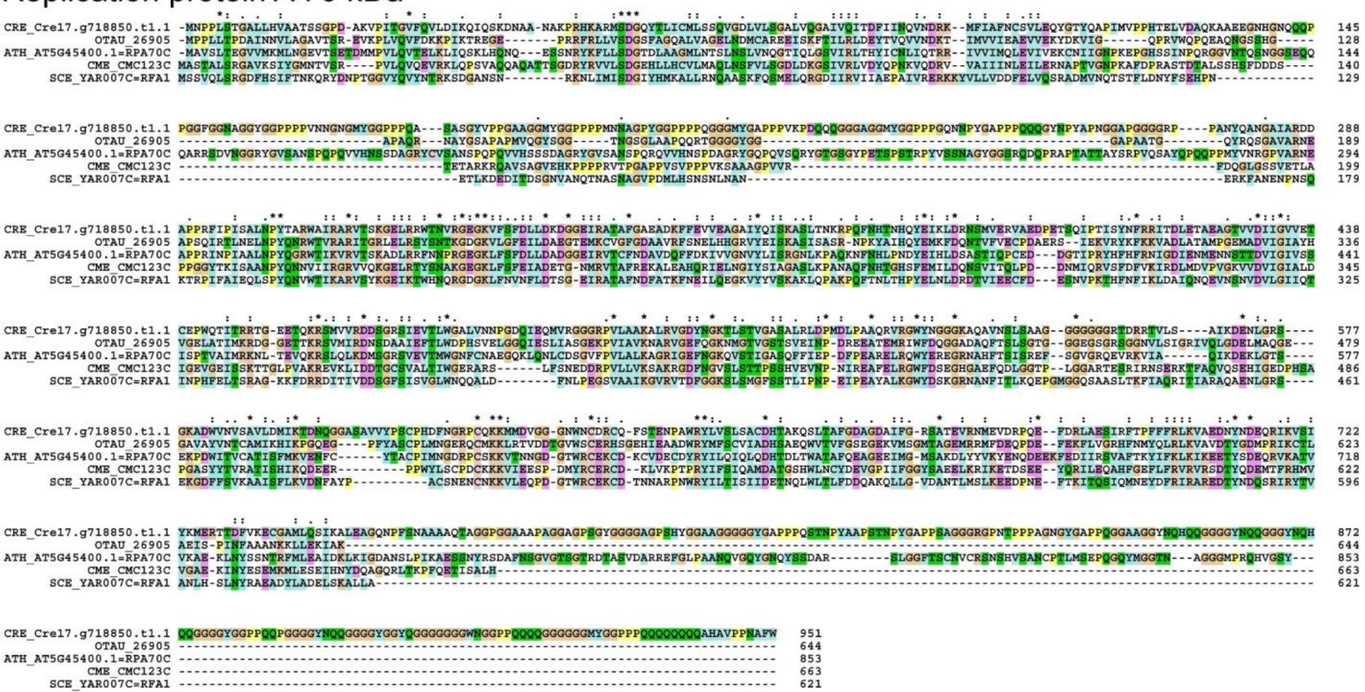

Supplementary figure S2-7

## Topoisomerase IV (subunit A)

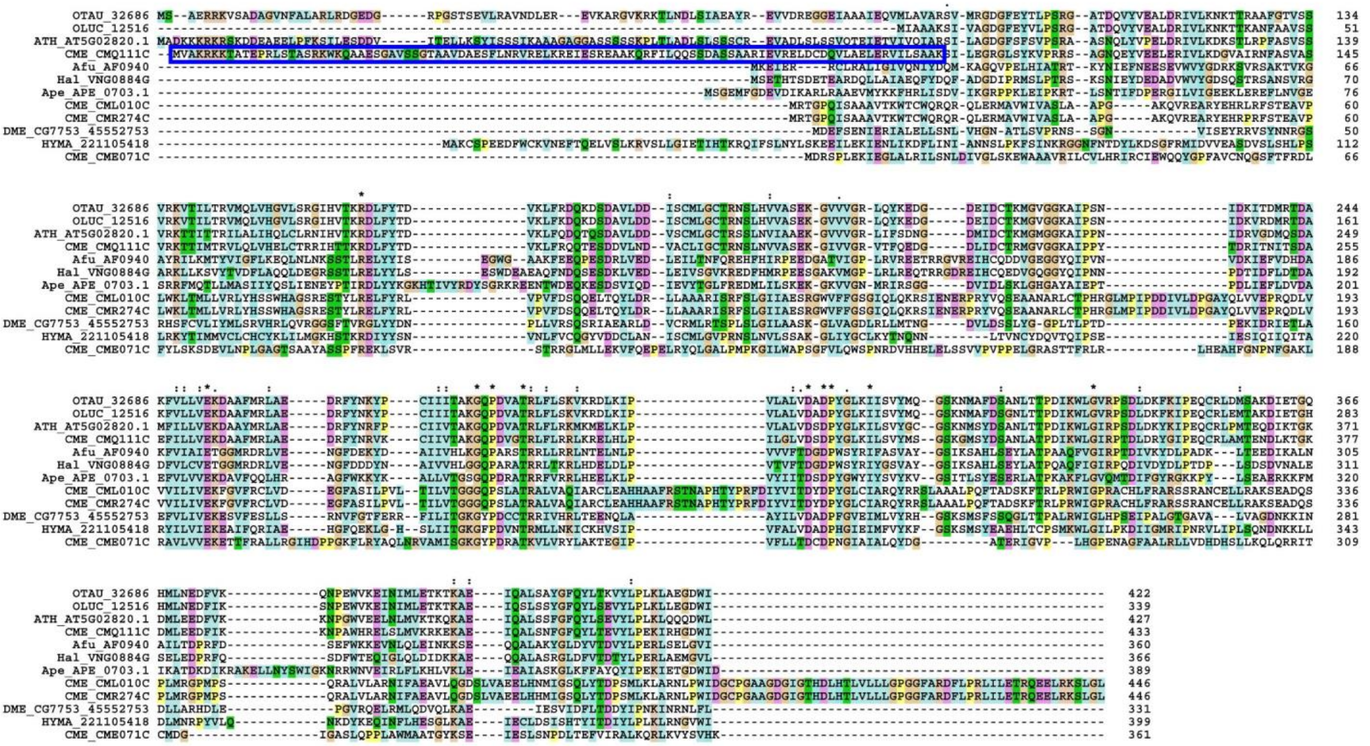

## Topoisomerase IV (subunit B)

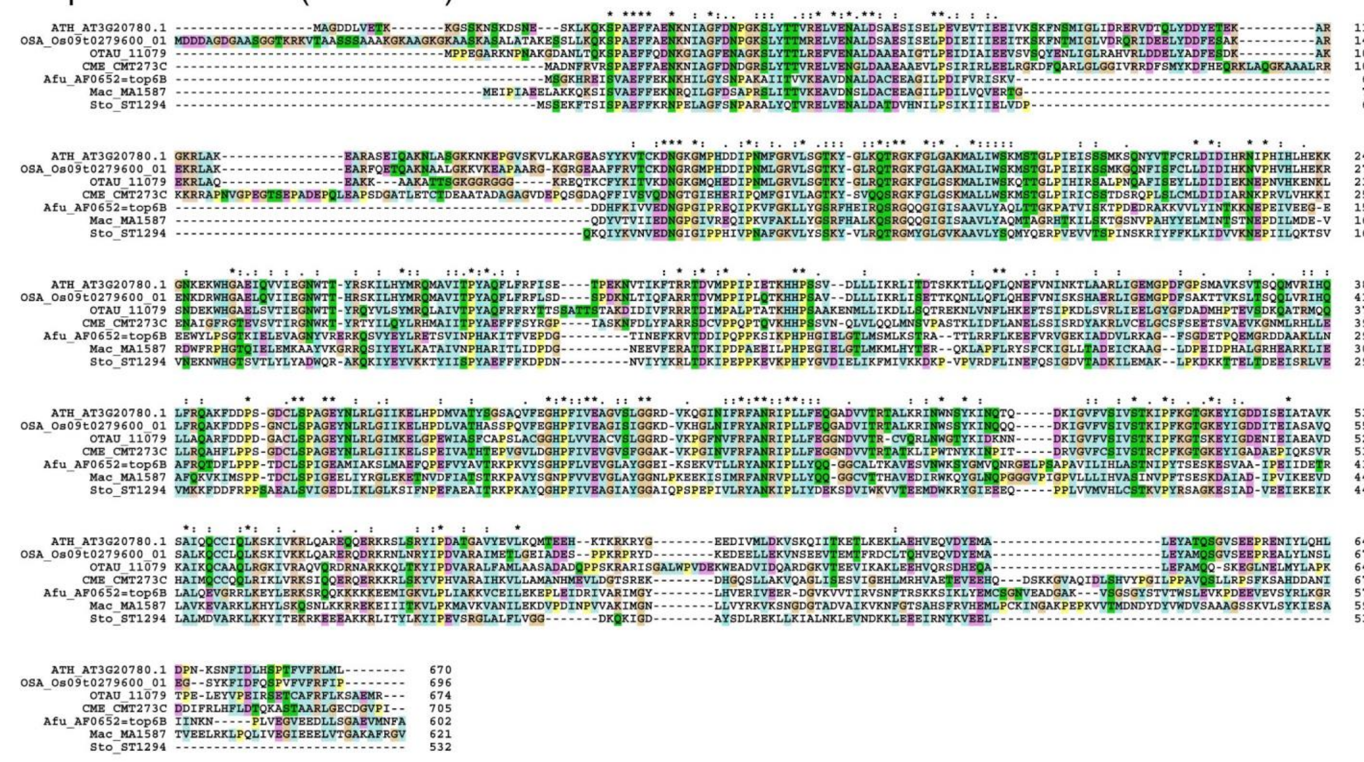

Supplementary figure S2-8

## DNA2

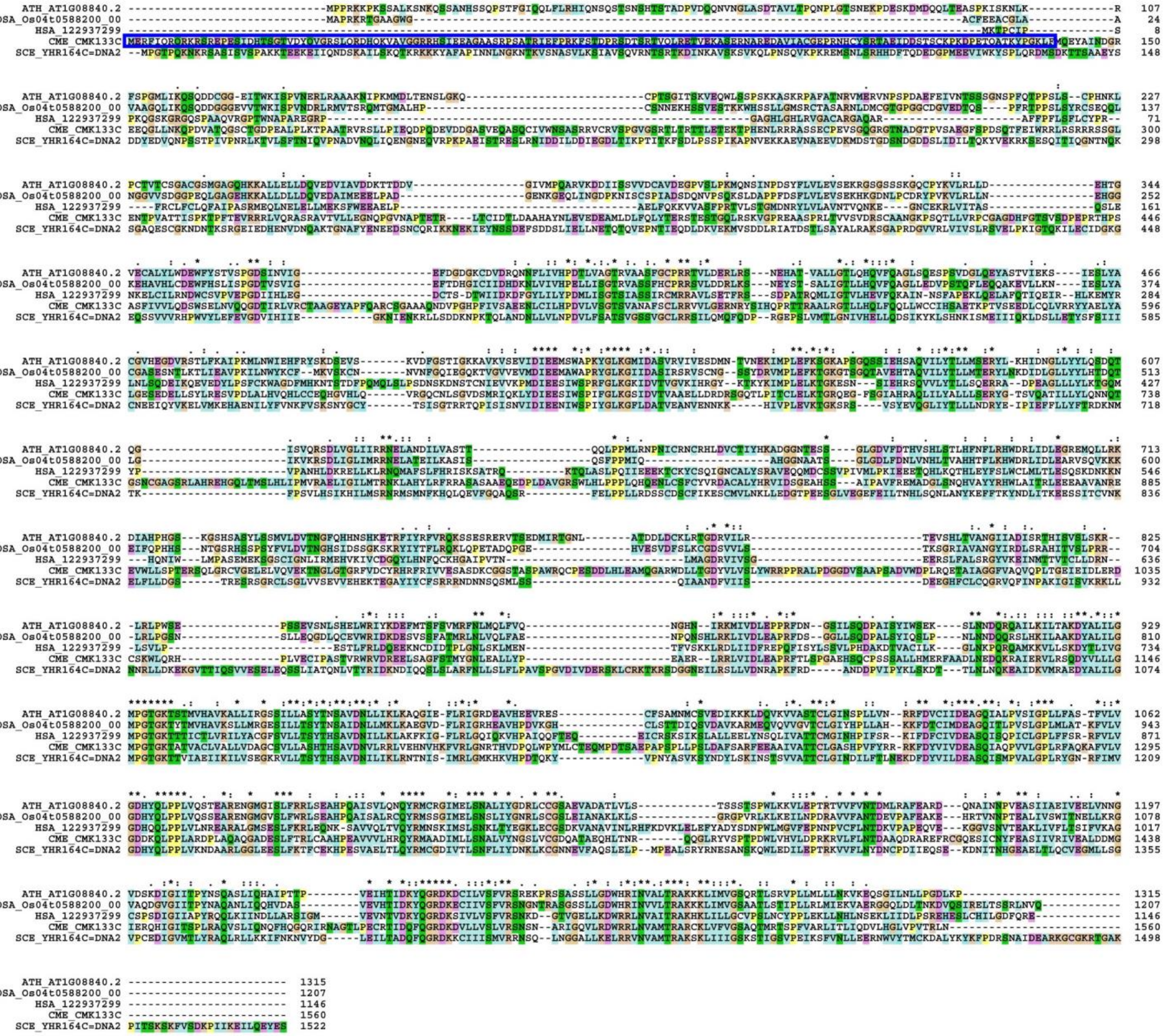


## FEN1

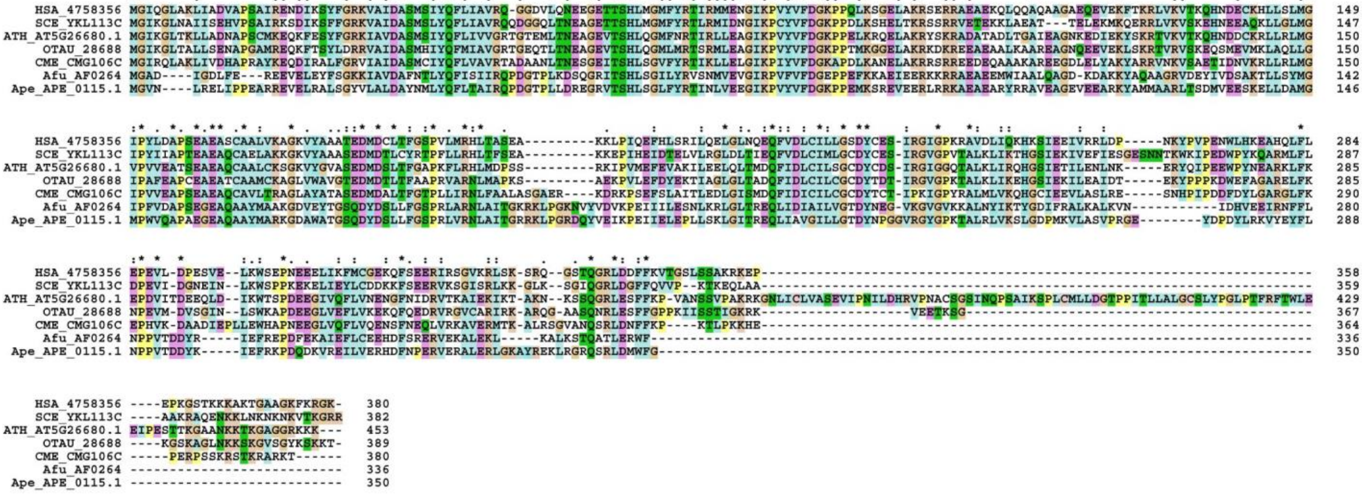


Supplementary figure S2-9

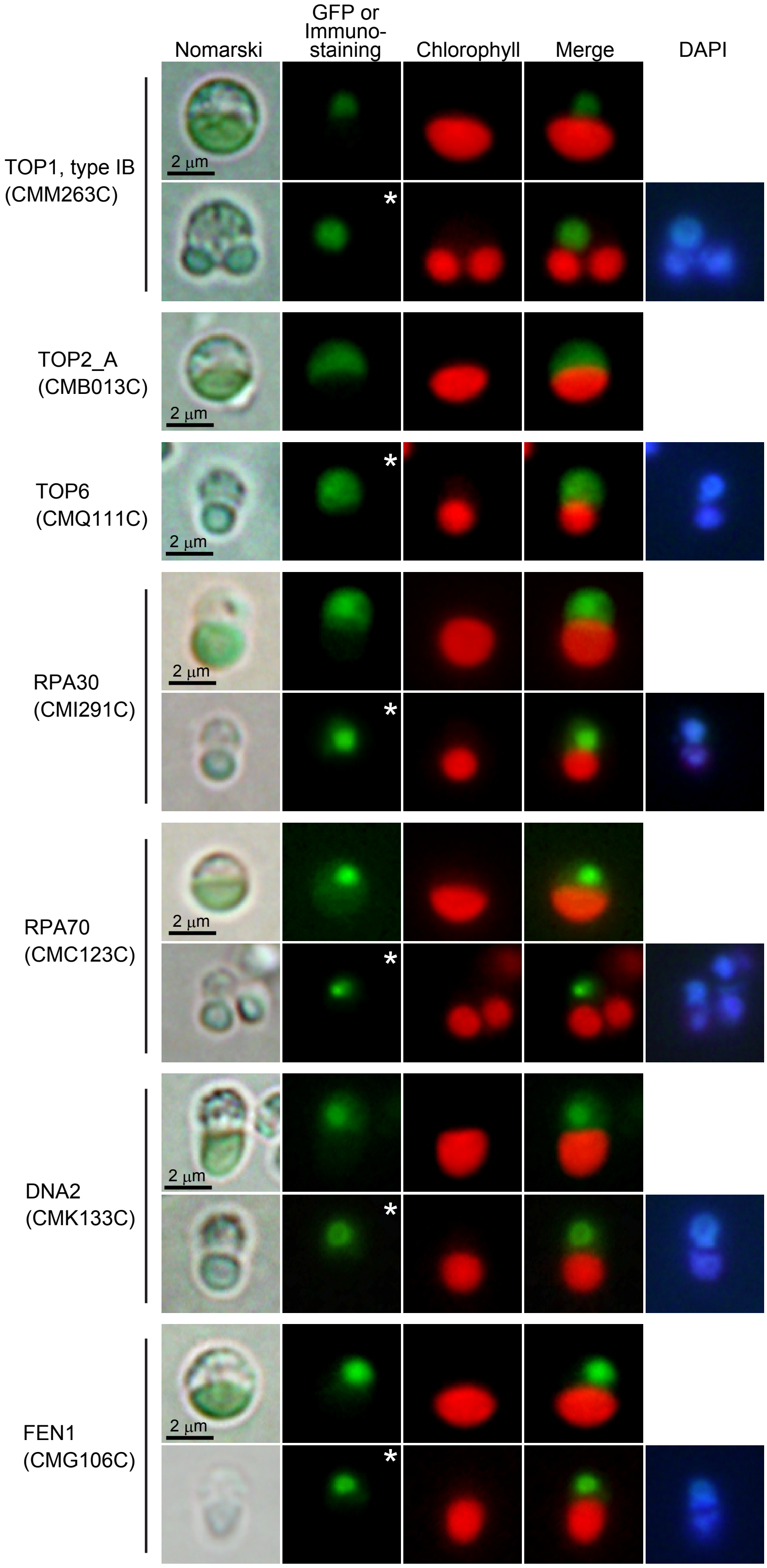


Supplementary Figure S3

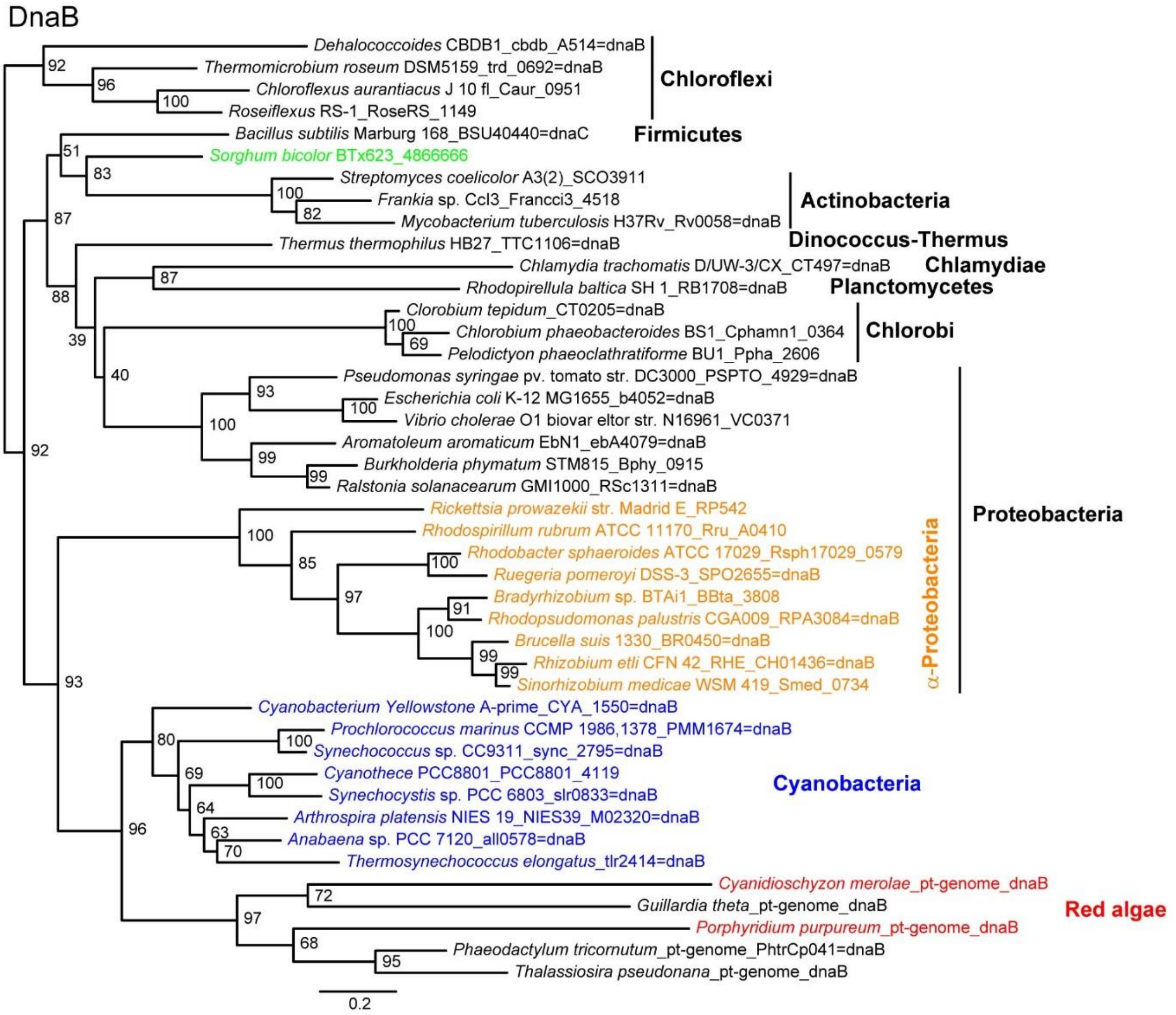


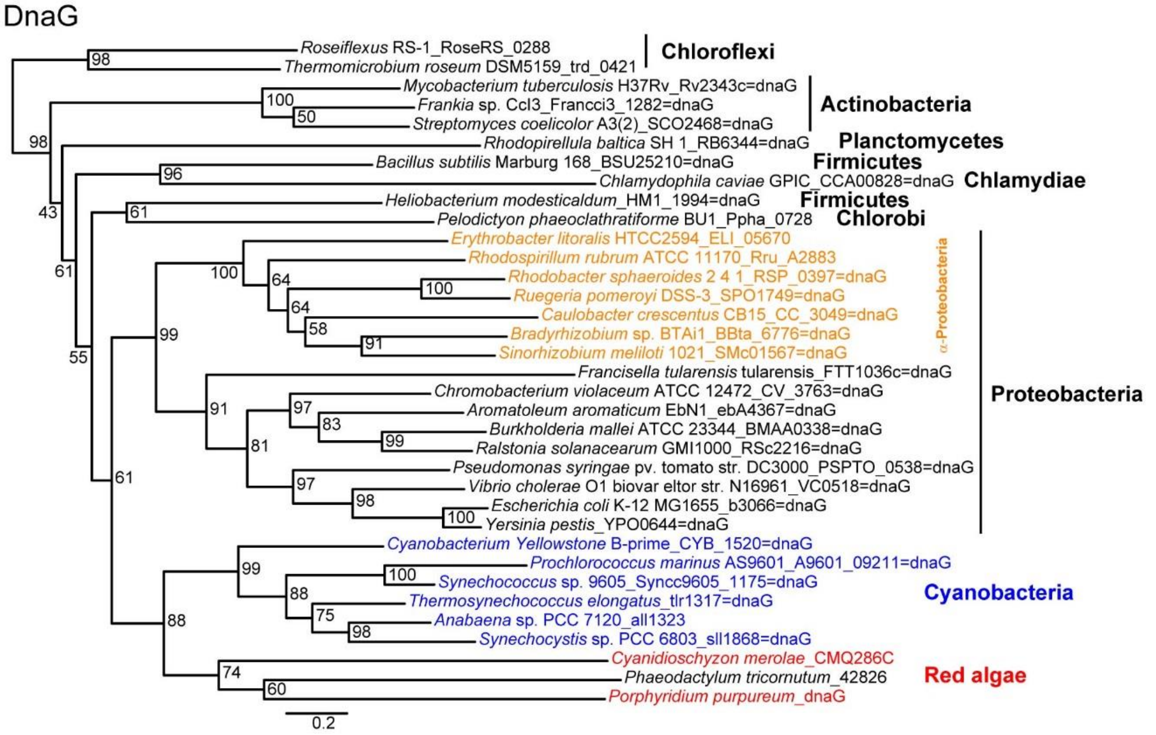


Supplementary figure S4-1

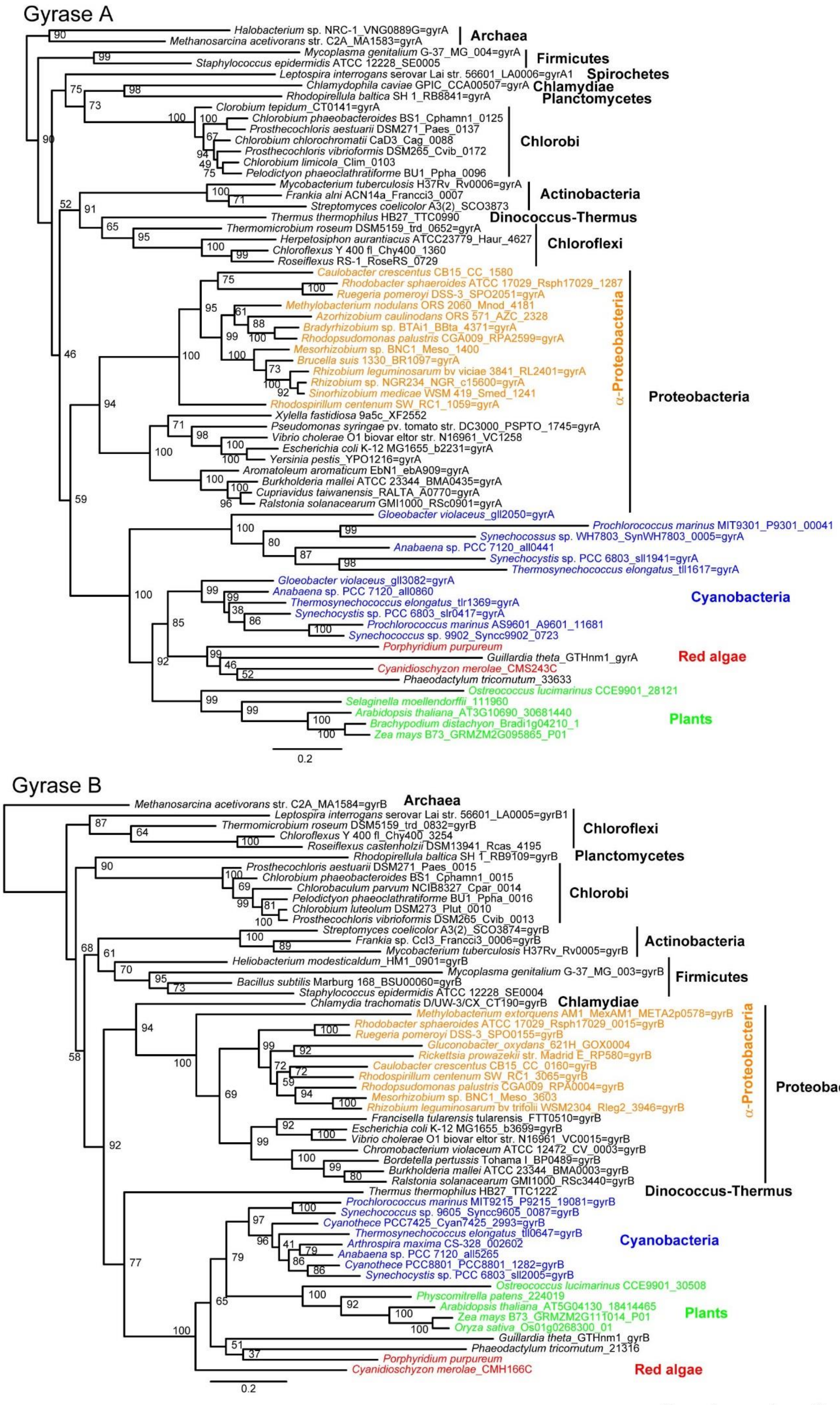


Supplementary figure S4-2

TOP1 (type IA topoisomerase)

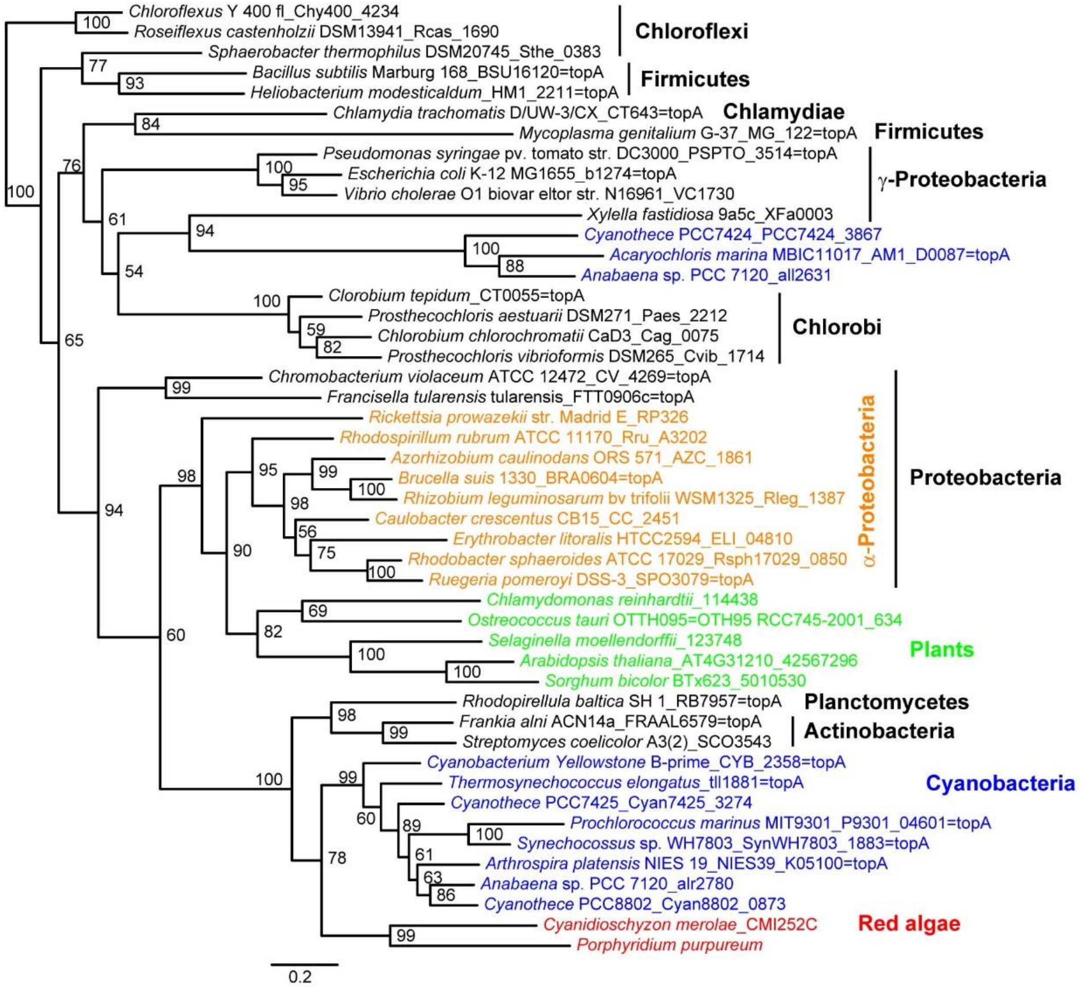


Supplementary figure S4-3

SSB

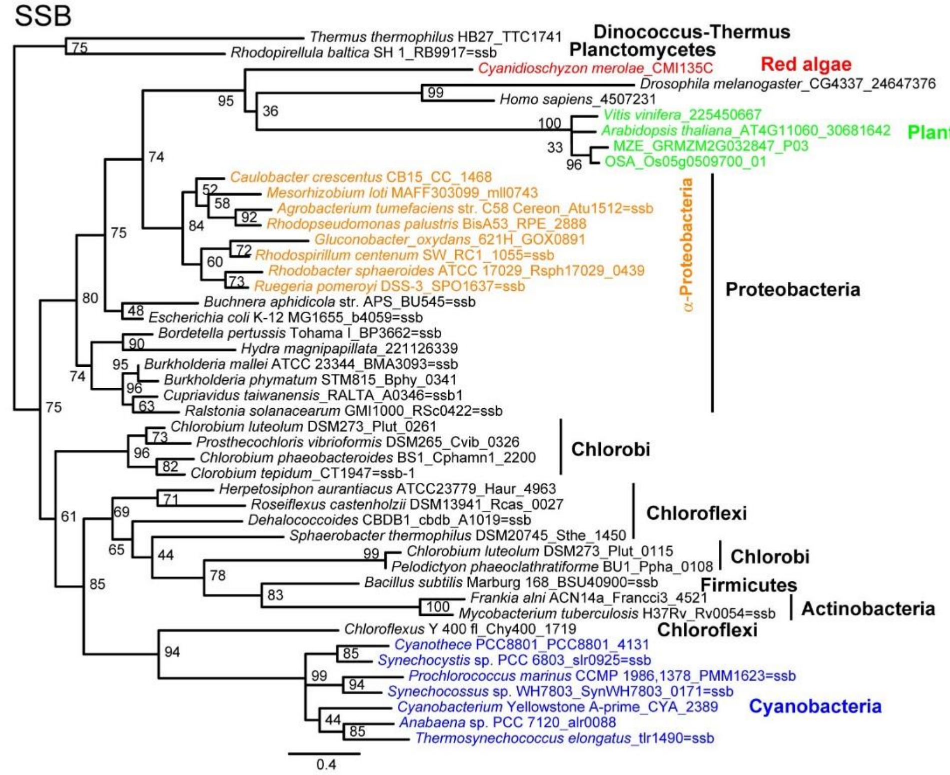


DNA polymerase I (5’ -3’ exonuclease domain)

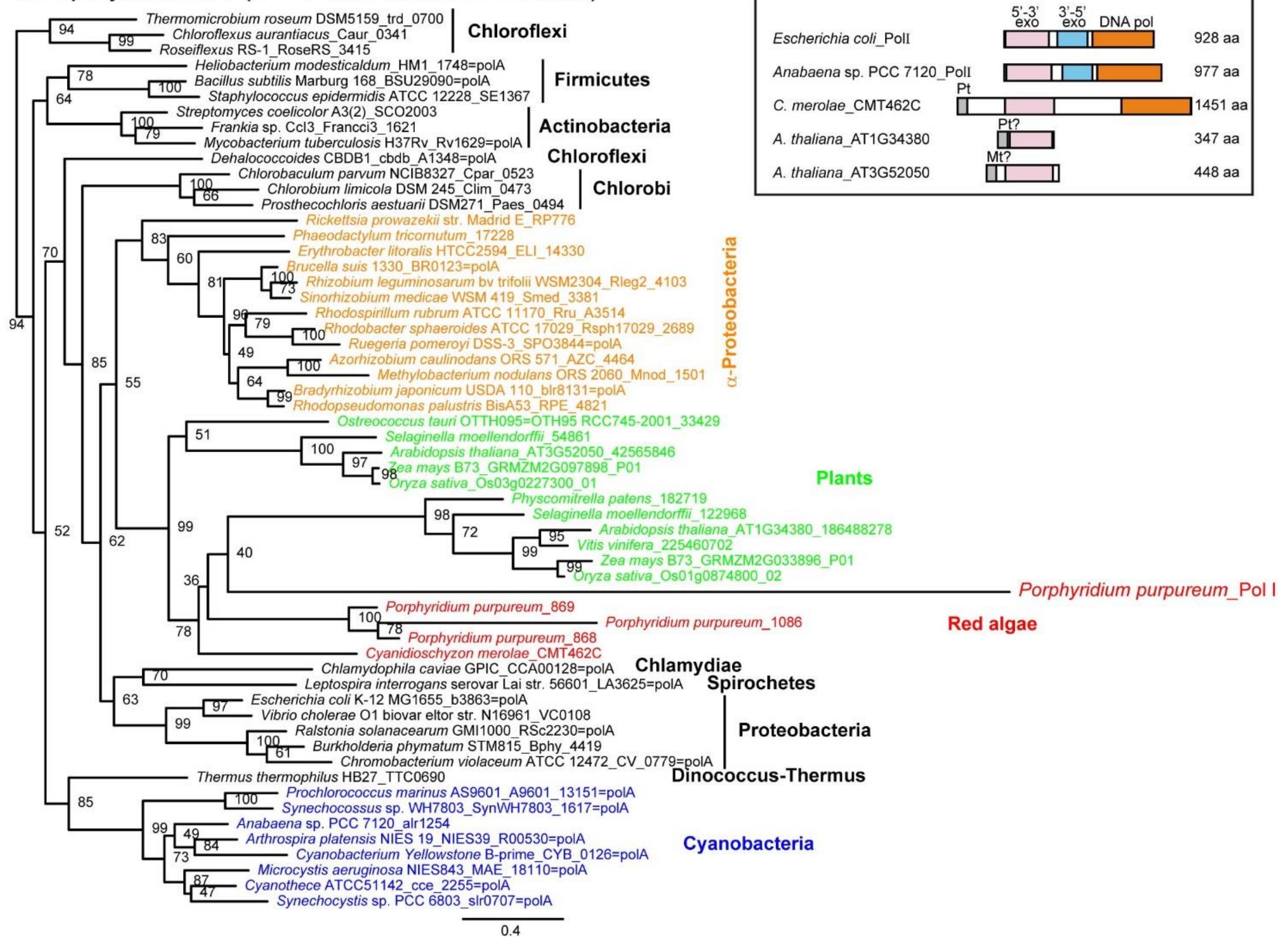


Supplementary figure S4-4

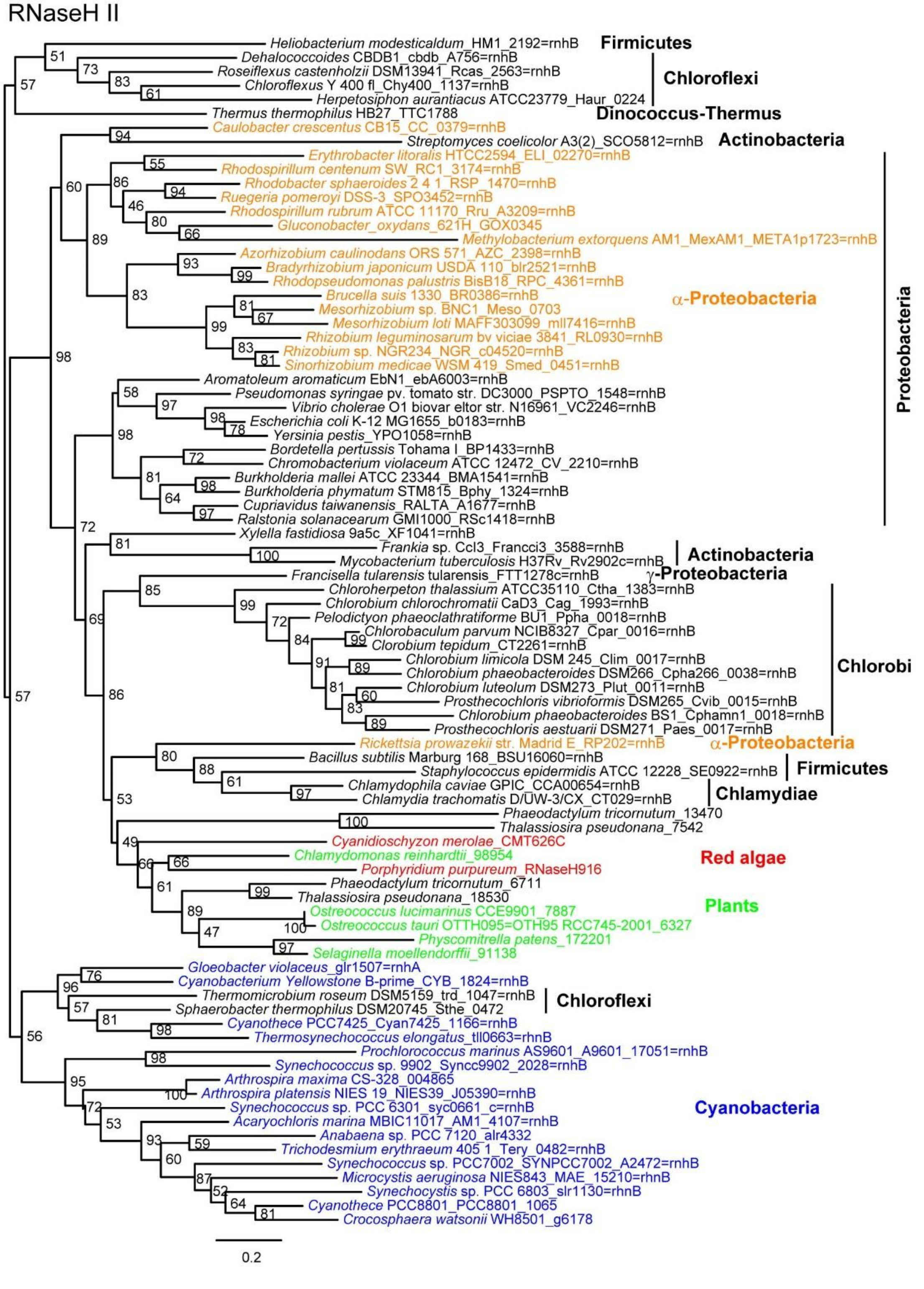


Supplementary figure S4-5

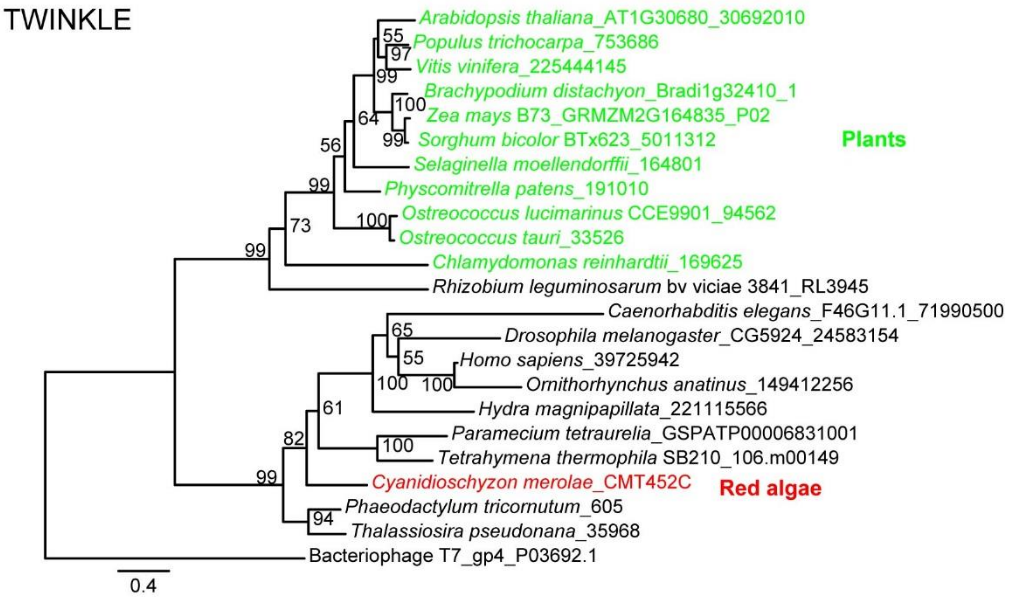


Supplementary figure S4-6
